# Genome-wide analysis of H4K5 acetylation associated with fear memory in mice

**DOI:** 10.1186/1471-2164-14-539

**Published:** 2013-08-08

**Authors:** C Sehwan Park, Hubert Rehrauer, Isabelle M Mansuy

**Affiliations:** 1 Department of Health Science and Technology, ETH Zürich, Zürich, Switzerland; 2 Functional Genomics Center of Zürich, Zürich, Switzerland; 3 Brain Research Institute, Medical Faculty of the University of Zürich, Zürich, Switzerland

**Keywords:** ChIP-Seq, Contextual fear conditioning, Gene bookmarking, Gene priming, H4K5 acetylation, Learning and memory

## Abstract

**Background:**

Histone acetylation has been implicated in learning and memory in the brain, however, its function at the level of the genome and at individual genetic loci remains poorly investigated. This study examines a key acetylation mark, histone H4 lysine 5 acetylation (H4K5ac), genome-wide and its role in activity-dependent gene transcription in the adult mouse hippocampus following contextual fear conditioning.

**Results:**

Using ChIP-Seq, we identified 23,235 genes in which H4K5ac correlates with absolute gene expression in the hippocampus. However, in the absence of transcription factor binding sites 150 bp upstream of the transcription start site, genes were associated with higher H4K5ac and expression levels. We further establish H4K5ac as a ubiquitous modification across the genome. Approximately one-third of all genes have above average H4K5ac, of which ~15% are specific to memory formation and ~65% are co-acetylated for H4K12. Although H4K5ac is prevalent across the genome, enrichment of H4K5ac at specific regions in the promoter and coding region are associated with different levels of gene expression. Additionally, unbiased peak calling for genes differentially acetylated for H4K5ac identified 114 unique genes specific to fear memory, over half of which have not previously been associated with memory processes.

**Conclusions:**

Our data provide novel insights into potential mechanisms of gene priming and bookmarking by histone acetylation following hippocampal memory activation. Specifically, we propose that hyperacetylation of H4K5 may prime genes for rapid expression following activity. More broadly, this study strengthens the importance of histone posttranslational modifications for the differential regulation of transcriptional programs in cognitive processes.

## Background

The formation of memory requires highly orchestrated gene expression programs for the establishment and the stabilization of memory traces over time. These programs are initiated during learning and can persist for several hours [1,2]. Whole genome expression studies have shown that some of these programs are needed for basal homeostatic cellular functions, while others are specific for cognitive functions [3-5]. The composition and regulation of transcriptional programs however may depend on the strength and duration of training. Its well known, for example, that practice or repeated training of a skill or concept can improve memory for the subject. Multiple training sessions required to form strong memory traces may, therefore, be associated with increased gene expression or the reinforcement of existing transcriptional programs, such as those necessary for structural changes to strengthen synaptic circuits [6-10]. How this is induced at the level of chromatin and which genes are targeted by epigenetic processes remains poorly understood.

With the emergence of the post-genomic era, recent studies in the field of learning and memory have investigated the implication of chromatin remodeling in cognitive processes. Several studies have revealed that chromatin remodeling plays a critical role in memory formation [9,11-14]. Chromatin remodeling is a complex molecular and structural process that involves the dynamic regulation of nucleosomes through different epigenetic mechanisms including histone posttranslational modifications (PTMs), DNA methylation and RNA interference [15-17]. In the rodent brain, several histone PTMs are rapidly induced and are associated with altered gene transcription following training. Acetylation of lysine 9 and 14 on H3 (H3K9ac, H3K14ac), of lysine 5, 8 and 12 on H4 (H4K5ac, H4K8ac, and H4K12ac), and of lysine 5, 12, 15, and 20 on H2B (H2BK5ac, H2BK12ac, H2BK15ac, and H2BK20ac), increases in the hippocampus following contextual fear conditioning (CFC) [4,11,18,19], a well-established behavioral paradigm for the establishment of contextual fear memory. Moreover, inhibition of histone deacetylases (HDACs) by HDAC inhibitors such as suberoylanilide hydroxamic acid (SAHA), sodium butyrate, valproic acid or trichostatin A can enhance memory and rescue deficits in contextual memory in rodents [4,12,20-25].

Although these studies provide strong evidence that histone acetylation is modulated by memory formation, a global assessment of histone acetylation at the level of the genome and the mechanism with which it regulates gene expression in memory processes is lacking. Using a genome-wide approach, we examined the distribution of H4K5ac, a mark of active chromatin implicated in transcriptional re-activation of post-mitotic cells through gene bookmarking [26], and its role in regulating transcriptional activity following the establishment of contextual fear memory in the adult mouse [4,13]. We propose that gene bookmarking may also be relevant in the hippocampus following learning, whereby genes may be primed for rapid induction through activity-induced histone acetylation. Using chromatin immunoprecipitation followed by deep sequencing (ChIP-Seq) and bioinformatics analysis, we show that H4K5ac in the hippocampus is prevalent throughout the genome and is a mark characteristic of actively transcribed genes. Motif analysis for conserved transcription factor (TF) binding sites (TFBS), however, reveal that gene expression depends on the enrichment of H4K5ac at consensus TFBS in the promoter and proximal to the TSS. We also identify a unique set of genes differentially acetylated for H4K5 and functionally associated with memory processes. Based on our findings, we propose a potential mechanism for priming genes through activity-dependent hyperacetylation of H4K5 in the promoter upon learning.

## Results

### Fear memory induces H4K5ac in the hippocampus in a training-dependent manner

**Figure 1:**
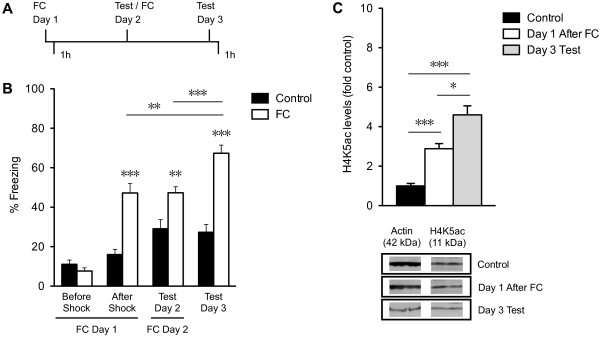
**Contextual fear memory and its association with H4K5ac in the hippocampus. (A)** Scheme of the behavioral experiment. Mice were trained on contextual fear conditioning (CFC) on day 1 (FC Day 1), tested 24 hours later on day 2 (Test Day 2), or conditioned again immediately following the test (FC Day 2), and tested again 24 hours later on day 3 (Test Day 3). Hippocampi were collected either one hour following FC Day 1 or one hour following Test Day 3. **(B)** Fear memory was measured as the freezing response before and after the foot-shock (shock) on FC Day 1, after re-exposure to the context on day 2 (Test Day 2), and after re-exposure to the context on day 3 in fear-conditioned (FC; n = 12) and in non-fear-conditioned controls (Control; n = 11). **(C)** Quantitative analysis and representative immunoblots of H4K5ac levels in nuclear fractions from whole hippocampus in controls one hour after context on day 1 (Control, n = 4), one hour after CFC on day 1 (Day 1 After FC; n = 4), or one hour after the memory test on day 3 (Day 3 Test; n = 3). Samples derived for immunoblots were processed in parallel using actin for normalization and run on two different gels. Error bars indicate SEM. *p < 0.05, **p < 0.01, ***p < 0.001.

To examine the epigenetic and transcriptional profile of genes associated with memory formation in the hippocampus, we trained adult mice on a CFC paradigm (Figure 1A). We chose CFC because it is a robust, long-lasting learning paradigm in which memory for a context can persist for more than one year after a single training session [27,28]. Mice were exposed to a novel context in which they received a foot-shock, either once (Day 1) or twice on two consecutive days (Day 1 and Day 2), then tested for fear memory 24 hours later (Day 3). After a single foot-shock, the animals expressed a significant freezing response (47.2 ± 16.5%; p < 0.001) compared to control mice (After Shock, FC Day 1; Figure 1B) that was maintained when tested 24 hours later (47.3 ± 10.0%; p < 0.01) (Test Day 2; Figure 1B). However, with a second training session on day 2, the freezing response was increased further by 20% (67.4 ± 14.2%; p <0.001) when tested 24 hours later (Test Day 3; Figure 1B). In control mice, freezing on days 2 and 3 compared to day 1 (Before Shock) was significant (p < 0.01 and p < 0.05, respectively), but was not significant compared to day 1 (After Shock), which is the measure by which we make all comparisons. It is also worth noting that control mice plateau on day 2 while FC mice continue to have higher freezing.

FC has been associated with transcriptional programs that are activated within 1 hour after conditioning, and that persist for up to 6 hours [3,29]. Subsequent training, however, may increase gene expression, recruit additional genes to reinforce the memory, or prime existing transcriptional programs for rapid induction of genes for synaptic strengthening. Since memory formation has been associated with histone acetylation in the brain, we examined whether memory performance correlates with higher acetylation levels following additional training sessions. We determined the level of H4K5ac, a PTM recently implicated in gene bookmarking, and increased with FC and object recognition memory tasks [4,13], following one or two days of CFC. Western blots show that H4K5ac was increased approximately 3-fold in the hippocampus 1 hour after one CFC session. With two conditioning sessions, H4K5ac level was increased 4.6-fold over controls following a memory test on day 3 (Figure 1C), suggesting that H4K5ac induction is proportionate to the amount of training. H4K5ac was examined 1 hour after memory test on day 3 because 1) gene expression is activated within 1 hour following fear conditioning and memory retrieval [30-32], 2) memory is consolidated or reconsolidated within 6 hours [3,29,32], 3) histone acetylation decreases to baseline levels within 2–4 hours [4,33], 4) memory for the context is enhanced by an additional training session, and 5) H4K5ac levels are higher at this time point.

### Distribution of H4K5ac across the genome and within genes

Previous studies have shown the association of histone acetylation at promoters of a restricted set of canonical genes involved in memory [4,9,13], but to date, genome-wide data are limited. Here, we used ChIP-Seq to determine the distribution of H4K5ac across the genome, followed by *de novo* identification of genes associated with H4K5ac after CFC (after 2 training sessions) in the mouse hippocampus.

**Figure 2:**
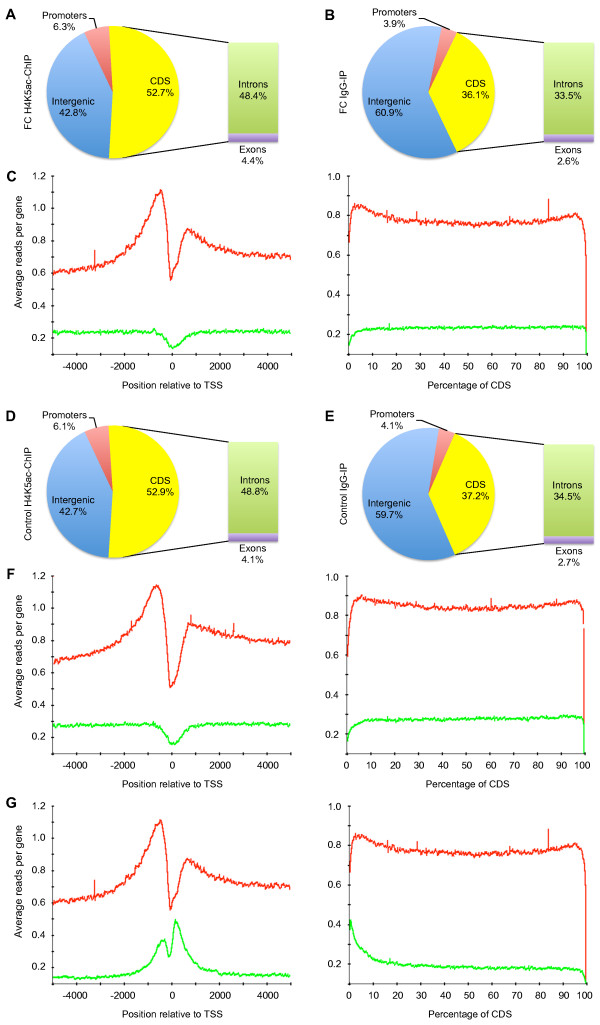
**Distribution of H4K5ac in the genome and its profile relative to the TSS. (A)** Distribution of reads from H4K5ac with respect to intragenic regions including promoter, CDS (introns and exons) and intergenic regions in the hippocampus after CFC compared to **(B)** mock IgG-IP. Promoters were defined as 5 kb upstream of the TSS, the CDS as regions between the TSS and TTS, and intergenic as regions excluding gene bodies. Reads spanning the transcription start site that match both a promoter and transcript are counted twice, thus, leading to total reads greater than 100%. **(C)** Profile of reads for H4K5ac (red) and IgG-IP controls (green) across ± 5 kb relative to the TSS (left) and spanning the CDS as a percentage of reads averaged over all genes (right). **(D)** Distribution of reads for H4K5ac and **(E)** IgG-IP by genomic regions in the absence of CFC (Control). **(F)** Profile of reads for H4K5ac (red) or IgG-IP (green) in Control. Distribution profiles for H4K5ac after 2 days CFC (red) compared to **(G)** H4K12ac immediately after 1 CFC session (green) [4]. Window analyses were obtained using EpiChIP [48].

Analysis of H4K5ac distribution showed enrichment of reads in the promoter and coding sequence (CDS) of H4K5ac-ChIP samples compared to IgG-IP samples in both FC (Figure 2A and 2B) and controls (Figure 2D and 2E), an increase of 19% (59 million read) and 17.7% (55 million reads), respectively. The targeted enrichment of H4K5ac to gene bodies is consistent with the proposed role of this PTM in transcriptional regulation. Analysis of H4K5ac in genic regions revealed higher acetylation upstream of the transcription start site (TSS), spanning the CDS and extending down to the transcription termination site (TTS) compared to IgG-IP samples (Figure 2C and 2F). Specifically, there was a prominent peak of H4K5ac in the promoter region approximately 800 bp upstream of the TSS, as well as in the CDS 1 kb downstream of the TSS. H4K5ac distribution was similarly enriched in the control group (Figure 2D), suggesting that learning does not change the overall profile of this PTM in the hippocampus. IgG-IP samples showed low coverage in both groups (Figure 2C and 2F) and, thus, are appropriate input controls for H4K5ac-ChIP sequence reads.

To determine whether the observed profile was specific for H4K5ac, we compared it with H4K12ac, another histone PTM associated with fear memory, from a publicly available dataset [4]. Although H4K5ac and H4K12ac datasets could not be directly compared due to the different CFC training protocols used, the increase of both H4K5ac and H4K12ac immediately following CFC and the higher levels of H4K5ac after two training sessions, suggest that histone acetylation is a consistent marker of memory formation. As with H4K5ac, our analysis of H4K12ac revealed a similar bimodal peak centered at the TSS which was restricted to approximately ± 1 kb relative to the TSS but did not extend into the CDS and TTS as with H4K5ac (Figure 2G). Moreover, H4K12ac had lower enrichment in the promoter than in the CDS, in contrast to H4K5ac, which was largely enriched in the promoter. We were unable to compare H4K12ac controls, as ChIP-Seq controls for sample and experimental conditions for H4K12ac were not available in the public release of this dataset. Together, these data suggest different occupancy and potentially different modes of transcriptional regulation by H4K5ac and H4K12ac following learning [34].

### H4K5ac as a marker of actively transcribed genes in the adult hippocampus

**Figure 3:**
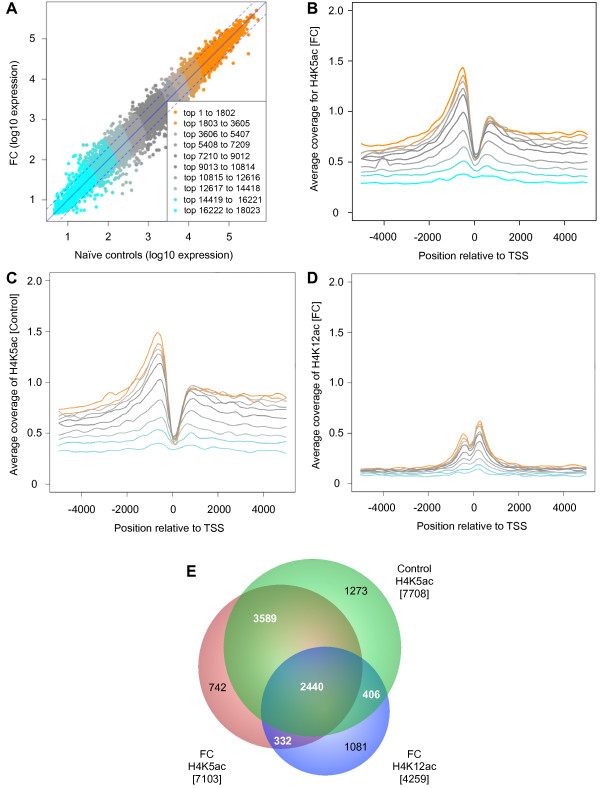
**Qualitative assessment of H4K5ac and gene expression. (A)** Expressed genes in the adult mouse hippocampus 1 hour after CFC compared to naïve controls are ranked from the lowest (cyan) to the highest expressed (orange) into 10 equal bins (1801 genes). Genes ranked by expression are correlated to average coverage for H4K5ac in **(B)** FC and **(C)** control mice re-exposed to the context on day 3, or to **(D)** H4K12ac immediately after CFC on day 1; average read coverage ± 5 kb relative to the TSS. **(E)** Venn diagram of unique and overlapping genes identified by acetylation above the average across all genes: with an H4K5ac threshold of greater than 50 reads in promoter, and a threshold of greater than 10 reads in promoter for H4K12ac.

We then examined the relationship between H4K5ac and gene transcription using a publicly available whole mouse genome microarray dataset (Agilent) for gene expression immediately after CFC in the mouse hippocampus [4]. We reasoned that because gene expression occurs within 1 hour of both memory consolidation and reconsolidation [3,29-32,35], this dataset was appropriate to determine the association between H4K5ac and global gene expression. The 18,023 genes form the expression dataset were ranked by level of expression (from lowest to highest) in FC compared to naïve controls (Figure 3A) and plotted against the average coverage of H4K5ac ± 5 kb relative to the TSS. The level of gene expression was found to correlate to H4K5ac enrichment such that the highest expressed genes had the highest coverage for H4K5ac, while the least expressed genes had the lowest coverage (Figure 3B and 3C; Additional file 1: Figure S3A and 3D). This applied to both groups regardless of training, suggesting that H4K5ac is a general feature of expressed genes. We also confirmed that H4K12ac correlated with the level of gene expression (Figure 3D; Additional file 1: Figure S3B). There was no correlation between gene expression and IgG-IP coverage (Additional file 1: Figure S1A and 1B). These results indicate a clear association between both H4K5ac and H4K12ac and gene expression.

We then identified genes acetylated above average and performed a cross-wise comparison between experimental groups. Based on the average promoter read count of 45 in our dataset, we considered genes with more than 50 reads in the promoter as above average. From a total of 23,235 genes in the dataset, 7,103 genes were identified in the FC group, and 7,708 genes in the control (Figure 3E). Using this criteria, 742 genes (15.1%) were specific for FC, 1,273 genes (21.8%) were specific for control, and 6,029 genes (85% of FC and 78% of control) were common to both groups. We then looked at whether genes with above average H4K5ac after 2 days of CFC were also associated with H4K12ac after one session of CFC. Using an adjusted threshold of 10 reads in promoter due to the lower average coverage, approximately 9 reads in promoter, in the H4K12ac dataset, we identified 4,259 unique genes with above average H4K12ac, of which 2,772 genes (65%) overlapped with genes with above average H4K5ac in FC, and 2,846 genes (67%) with above average H4K5ac in controls (Figure 3E; Additional file 1: Table S1). 2,440 genes overlapped all three groups using this criteria.

The results of these analyses extend our findings that in control conditions most nucleosomes are not only acetylated for H4K5 above the average of all genes, but are also acetylated for H4K12. Interestingly, nearly two-thirds of genes with above average H4K12ac after one session of CFC was found to overlap with above average H4K5ac after 2 days of CFC or context. This suggests that the same set of genes, associated with H4K12ac and induced immediately after CFC, may be upregulated following reinforced training, regardless of the associated histone acetylation used to identify the genes. It also suggests that the same set of genes may be activated after initial learning, during the formation of contextual fear memory, and after memory retrieval, independently of the CFC paradigm.

### H4K5ac is associated with both promoter and coding regions

**Figure 4:**
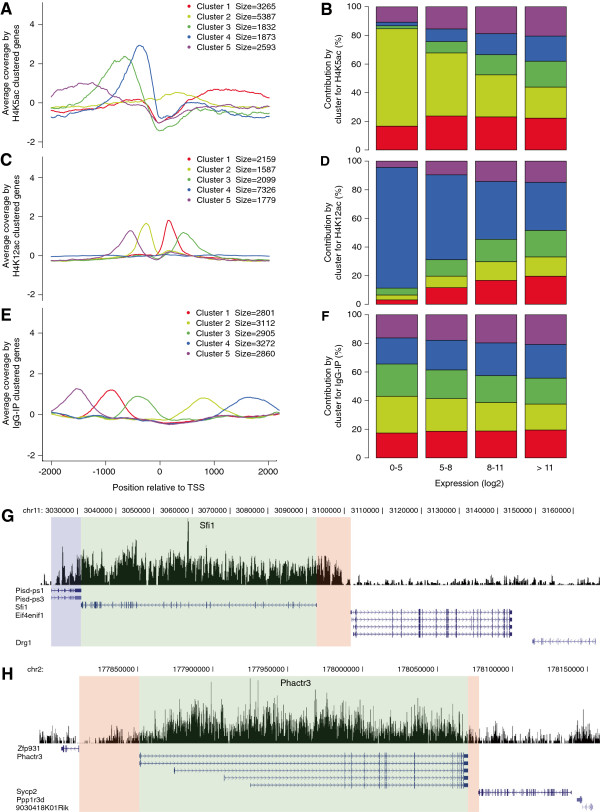
**H4K5ac enrichment in the promoter and CDS. (A)** Genes acetylated for H4K5 after CFC are clustered based on acetylation profiles over ±2 kb relative to the TSS. Clusters 3 (green), 4 (blue), and 5 (violet) occur in promoter regions, cluster 1 (red) occurs in the CDS, and cluster 2 (yellow) is unenriched for H4K5ac. **(B)** Contribution of gene clusters with respect to levels of gene expression show higher contribution of clusters enriched for H4K5ac with higher expression. **(C)** Clustering of genes acetylated for H4K12ac after CFC generated clusters 2 (yellow) and 5 (violet) in the promoter region, clusters 1 (red) and 3 (green) in the CDS, and cluster 4 (blue) is unenriched for H4K12ac. **(D)** The contribution of clusters enriched for H4K12ac is also higher with higher gene expression levels. **(E)** Clustering of genes obtained from IgG-IP controls after CFC result in uniformly distributed clusters including clusters 1 (red), 3 (green), and 5 (blue) in the promoter region, and clusters 2 (yellow) and 4 (blue) in the CDS. **(F)** IgG clusters show no change in contribution with increasing gene expression levels. **(G, H)** Custom tracks are shown for **G)***Sfi1* (chr11:3073466–3095466) and **H)***Phactr3* (chr2:177908053–177930053), as identified by both MACS and SICER for genes differentially acetylated for H4K5 in FC over control. A gene contiguous to *Sfi1* (Pisd-ps1/3) is highlighted in blue, the CDS is highlighted in green, and adjacent intergenic regions in pink. Neighboring genes (*Eif4enif1*, *Drg1*, *Zfp931*, *Sycp2*, *Ppp1r3d*, and *9030418K01Rik*) are shown to emphasize H4K5ac enrichment in the vicinity of peak-called genes. Splice variants for *Phactr3* (Refseq) are annotated below the track. Customized wig tracks were visualized in the UCSC Genome Browser.

Nucleosome occupancy studies have shown that acetylated and methylated histones are enriched in the promoter of highly expressed genes, but subsequently removed or replaced in the CDS [36-38]. To investigate the positional effect of nucleosomes with H4K5ac on transcription, we clustered genes based on their acetylation profile ± 2 kb relative to the TSS. Five H4K5ac clusters were identified in FC: one in the CDS (cluster 1), one with relatively no enrichment (cluster 2), and three in the promoter (clusters 3, 4, and 5) (Figure 4A). Genes with H4K5ac that feature in either the promoter or the CDS (clusters 1 and 3–5) constituted a larger proportion of highly expressed genes, while genes with relatively no enrichment (cluster 2) accounted for the largest proportion of genes with low expression (Figure 4B). Genes clustered for H4K5ac in controls had profiles and cluster contributions relative to expression comparable to FC (Additional file 1: Figure S2A and 2B). For H4K12ac-clustered genes, we obtained two in the promoter (clusters 2 and 5) and two in the CDS (clusters 1 and 3), which contributed to a greater proportion of highly expressed genes compared to the non-enriched cluster (cluster 4) (Figure 4C and 4D). In contrast, IgG-IP-clustered genes, which were not enriched for H4K5ac, had equal distribution in low, moderate, and highly expressed genes, regardless of training or the histone mark (Figure 4E and 4F; Additional file 1: Figure S2C and 2D). Promoter, CDS, and 3’-UTR-associated genes correlated with H4K5ac and H4K12ac, with and without CFC, but did not correlate with IgG-IP clusters (Additional file 1: S3A and 3E).

These findings suggest that H4K5ac in the promoter and/or CDS may be a feature of highly expressed genes. To validate this observation, we examined the profile of H4K5ac in *Sfi1* and *Phactr3*, two representative genes differentially acetylated for H4K5ac in CFC and involved in cell division in mitotic cells and in memory processes [39,40], respectively (Additional file 1: Table S2). In *Sfi1*, *Phactr3*, and *Phactr3* splice variants*,* H4K5ac was targeted specifically to the CDS (green) (Figure 4G and 4H). For *Sfi1*, H4K5ac was also highly enriched in the adjacent CDS of *Pisd-ps1/3* (blue; Figure 4G), and downstream of the TTS in an intergenic region preceding the CDS of *Eif4enif1* (pink; Figure 4G). In contrast, the CDS of *Eif4enif1* and *Drg1* showed dramatically lower H4K5ac. The overlap of H4K5ac in the CDS of *Sfi1* and *Pisd-ps1/3* translated to similar expression levels for *Sfi1* (15.19; shown as log2 expression and hereafter) and *Pisd-ps1/3* (14.72) but not for *Eif4enif1* (11.48) or *Drg1* (12.44), which had lower enrichment for H4K5ac. For *Phactr3*, H4K5ac coverage was lower in intergenic and CDS of neighboring genes *Zfp931*, *Sycp2*, and *Ppp1r3d* (pink; Figure 4H). The effect of H4K5ac on gene expression was also clearly evident for *Phactr3* (15.07) and neighboring genes, *Zfp931* (11.42), *Sycp2* (3.97), and *Ppp1r3d* (11.51), which show lower expression levels. This provides further evidence that the level of H4K5ac enrichment in the CDS is directly proportional to the level of gene transcription.

### TF binding sites proximal to the TSS increase the statistical probability of H4K5ac-nucleosome occupancy in the promoter

**Figure 5:**
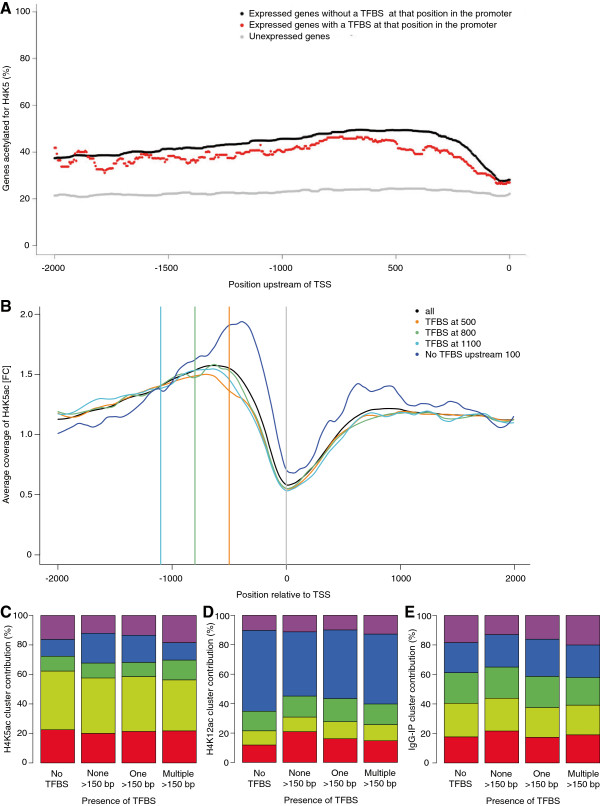
**H4K5ac in the presence of TF binding sites relative to the TSS. (A)** Percent of expressed genes acetylated for H4K5 by CFC with (red) or without (black) a TFBS at that position in the promoter, 2 kb upstream of the TSS in 5 bp increments. Lines are smoothed with a running median of width 10. Given that a gene can be acetylated at multiple sites in the promoter regardless of whether it is expressed or not, the percentage of acetylated genes at a specific position can be greater than 100%. Percent of unexpressed genes that are acetylated are plotted in gray. **(B)** Average read coverage of H4K5ac by CFC, ± 2 kb relative to the TSS, for all genes (black), genes with a TFBS present 500 bp (orange), 800 bp (green), or 1100 bp (turquoise) upstream of the TSS, and genes with no TFBS upstream of 100 bp of the TSS (blue). The relative contribution of gene clusters for **(C)** H4K5ac by CFC, **(D)** H4K12ac by CFC, and **(E)** mock IgG-IP with either no TFBS present in the promoter, or no TFBS, one TFBS, and multiple TFBS present upstream of 150 bp relative to the TSS. Clusters are defined in Figures 4A, 4C, and 4E, respectively.

We next examined whether high levels of gene expression associated with H4K5ac is linked to permissible TF binding. We scanned the promoter region 2 kb upstream of the TSS for conserved TFBS, and computed the percentage of expressed genes with H4K5ac at that position (Figure 5A). For expressed genes, the percentage of acetylated genes was significantly lower across all positions with a consensus TFBS compared to positions without a known TFBS. Unexpressed genes accounted for approximately 20% of genes with H4K5ac. Our assumption is that having a TFBS at a specific position, on average, increases the probability that TF binding occurs at that position relative to a random sequence position in the presence of H4K5ac. To refine our search and identify regions in the promoter where TF binding may affect H4K5ac occupancy, we profiled the coverage of H4K5ac on all genes, on genes with a TFBS at 500 bp, 800 bp or 1100 bp upstream of the TSS, and on genes with no TFBS 100 bp upstream of the TSS (Figure 5B). Using the average coverage of H4K5ac of all genes as baseline, we observed that the presence of a TFBS at position −500 bp or −800 bp, and −1100 bp resulted in modest a reduction in H4K5ac relative to baseline coverage at that position. However, genes with no TFBS upstream of 100 bp resulted in significantly higher H4K5ac in both the promoter and CDS, approximately ±1 kb relative to the TSS.

Based on the increase of H4K5ac coverage in the absence of a TFBS upstream of 100 bp, we focused our analysis in this region, proximal to the TSS. We compared the contribution of acetylated gene clusters (Figure 4A, 4C, 4E; Additional file 1: Figure S2A and 2C) in the presence or absence of a TFBS relative to 150 bp of the TSS: either no TFBS present in the promoter (within 2 kb of the TSS) or no TFBS, one TFBS, or multiple TFBS 150 bp upstream of the TSS (Figure 5C-E; Additional file 1: Figure S4A and 4B). Gene clusters with relatively no enrichment for H4K5ac or H4K12ac constituted a larger proportion of genes regardless of whether a TFBS was present or not (cluster 2, yellow; Figure 5C; cluster 4, blue; Figure 5D). However, in the presence of at least one TFBS within 150 bp of the TSS (no TFBS > 150), the contribution of cluster 4 for H4K5ac in FC (blue, nearest the TSS in the promoter; Figure 4A and 5C), cluster 3 for H4K5ac in control (green, nearest the TSS in the promoter; Additional file 1: Figure S2A and 4A), and cluster 1 for H4K12ac after CFC (red, nearest the TSS in the CDS; Figure 4C and 5D) increased from approximately 10% to 20%, compared to the same clusters when no TFBS was present. To a lesser extent, cluster contribution was also increased in the presence of one TFBS 150 bp upstream of the TSS, but was diminished in the presence of multiple TFBS. These observations provide novel insight into H4K5ac-mediated regulation of gene transcription and support the notion that TF binding and acetylation are mutually exclusive in the promoter [41]. However, H4K5ac is increased when TF binding occurs proximal to the TSS.

The observed increase in acetylation and transcription at proximal TFBS may be attributed to the recruitment of transcriptional machinery including TFs and RNA polymerase II, which is also known to occupy positions near the TSS in actively transcribed genes [42]. Additionally, recent ENCODE studies have shown that a set of TFs is strongly associated to positions proximal to the TSS and that transcriptional initiation is determined by stereotyped TF binding in this region, approximately 100 to 200 bp upstream of the TSS [43,44]. Acetylated nucleosomes further away in the promoter, greater than 1 kb from the TSS, may either be more strongly bound and less easily displaced by TF binding, or they may be regulatory regions which do not depend on the presence or acetylation of nucleosomes [45]. As expected, IgG-IP control clusters were uniformly proportioned in the presence or absence of a TFBS (Figure 5E; Additional file 1: Figure S4B). Together, these data suggest that since H4K5ac is associated with increased gene expression, enrichment of H4K5ac proximal to the TSS may be a reliable marker of actively transcribed genes.

### Genes differentially acetylated for H4K5 are associated with fear memory in the hippocampus

The high percentage of genes with above average H4K5ac in both FC and controls suggest that this modification is important and that it is subject to tight regulation in the context of transcription-dependent memory formation. Using a criteria-based approach, we found that ~15% of genes were uniquely acetylated for H4K5 with CFC (Figure 3E), however, this did not account for differentially acetylated genes. We also found that H4K5ac correlates to global gene expression levels. Thus, to identify specific genes induced by learning and increased H4K5ac levels in the hippocampus, we used a top-down approach – rather than identifying specific genes activated by learning through differential gene expression, we identified highly expressed genes through differential acetylation of H4K5 in FC compared to controls. We used a peak-calling algorithm to scan the genome at 300 bp intervals for differentially acetylated regions between FC and controls. Using model-based analysis of ChIP-Seq (MACS), we obtained consensus coverage of H4K5ac-enriched regions across the mouse genome [46]. Out of 20,238 peaks identified for H4K5ac in FC by MACS, 708 peaks were found −4000 to −2000 bp relative to the TSS, 3,370 peaks were found in the promoter (−2000 to 0 bp), and 1,340 peaks were found in the CDS (0 to +2000 bp). Of these, we identified 241 regions significantly acetylated for H4K5 in FC, 115 of which were associated with gene bodies representing 114 unique genes, and 126 within intergenic regions (Additional file 1: Table S2).

To validate the results obtained with MACS, we repeated the analysis with three other published algorithms for ChIP-Seq analysis, including SICER, EpiChip, and Genomatix NGS analyzer (Additional file 1: Figure S5A – 5D) [47-49]. We performed a cross-wise comparison of genes identified with the algorithms to genes identified using pre-defined criteria, including genes with more than 50 reads in the promoter (Raw H4K5), previously defined as above average, or genes with more than 50 reads in the promoter with CFC but 40 reads or less in controls (Diff H4K5), analogous to algorithm-based differential acetylation (Figure 3E; Additional file 1: Figure S5E; see Methods). Of all genes identified by MACS, approximately 70% overlapped with SICER, the other most widely used algorithm for differential peak finding. Thus, we considered the genes identified by MACS as a reliable and representative gene set to evaluate further.

### Genes differentially acetylated for H4K5 in FC are associated with memory processes

Gene ontology analysis of the 114 unique MACS-derived genes in FC identified genes enriched for structural and neuronal components including synapses, the postsynaptic density, and axons, in addition to genes involved in functional processes such as synapse assembly and organization, ion transport, calcium signaling, neuromuscular and neurological system processes (Table 1; Additional file 1: Table S2 and 4). From interaction maps, we also found that genes in pathways involved in calcium, mTOR, Erbb signaling, and Alzheimer’s disease were significantly enriched (Table 2).

**Table 1:** GO enrichment analysis of MACS-identified genes differentially acetylated for H4K5 in FC and control in hippocampus

**GO term**	**Annotation**	**Treatment**	**Fold-change**	*P*-value*
GO:0045202	Cellular component: synapse	FC	5.78	8.01E-06
GO:0000267	Cellular component: cell fraction	FC	3.48	7.33E-05
GO:0005624	Cellular component: membrane fraction	FC	3.76	0.0001
GO:0003008	Biological process: system process	FC	3.03	0.0002
GO:0015491	Molecular function: cation:cation antiporter activity	FC	26.05	0.0002
GO:0005626	Cellular component: insoluble fraction	FC	3.59	0.0002
GO:0022892	Molecular function: substrate-specific transporter activity	FC	2.81	0.0004
GO:0022891	Molecular function: substrate-specific transmembrane transporter activity	FC	2.93	0.0004
GO:0014069	Cellular component: postsynaptic density	FC	11.65	0.0004
GO:0007416	Biological process: synapse assembly	FC	18.89	0.0005
GO:0006897	Biological process: endocytosis	FC	5.74	0.0006
GO:0010324	Biological process: membrane invagination	FC	5.74	0.0006
GO:0050885	Biological process: neuromuscular process controlling balance	FC	16.85	0.0007
GO:0065008	Biological process: regulation of biological quality	FC	2.28	0.0008
GO:0050877	Biological process: neurological system process	FC	3.08	0.0008
GO:0022857	Molecular function: transmembrane transporter activity	FC	2.74	0.0008
GO:0042592	Biological process: homeostatic process	FC	2.85	0.0009
GO:0006810	Biological process: transport	FC	1.84	0.001
GO:0043062	Biological process: extracellular structure organization	FC	6.49	0.001
GO:0030424	Cellular component: axon	FC	6.38	0.0011
GO:0005583	Cellular component: fibrillar collagen	FC	39.19	0.0011
GO:0044456	Cellular component: synapse part	FC	4.92	0.0014
GO:0005246	Molecular function: calcium channel regulator activity	FC	32.07	0.0017
GO:0015075	Molecular function: ion transmembrane transporter activity	FC	2.73	0.0021
GO:0015298	Molecular function: solute:cation antiporter activity	FC	11.8	0.0021
GO:0005794	Cellular component: Golgi apparatus	FC	2.84	0.0025
GO:0015385	Molecular function: sodium:hydrogen antiporter activity	FC	26.05	0.0026
GO:0005451	Molecular function: monovalent cation:hydrogen antiporter activity	FC	26.05	0.0026
GO:0005215	Molecular function: transporter activity	FC	2.24	0.0032
GO:0005581	Cellular component: collagen	FC	19.59	0.0046
GO:0000166	Molecular function: nucleotide binding	Control	4.43	5.04E-06
GO:0032553	Molecular function: ribonucleotide binding	Control	4.31	7.57E-05
GO:0032555	Molecular function: purine ribonucleotide binding	Control	4.31	7.57E-05
GO:0017076	Molecular function: purine nucleotide binding	Control	4.15	0.0001
GO:0005524	Molecular function: ATP binding	Control	4.76	0.0001
GO:0032559	Molecular function: adenyl ribonucleotide binding	Control	4.71	0.0001
GO:0005003	Molecular function: ephrin receptor activity	Control	112.59	0.0001
GO:0050771	Biological process: negative regulation of axonogenesis	Control	90.12	0.0002
GO:0021955	Biological process: central nervous system neuron axonogenesis	Control	96.56	0.0002
GO:0001883	Molecular function: purine nucleoside binding	Control	4.47	0.0002
GO:0030554	Molecular function: adenyl nucleotide binding	Control	4.49	0.0002
GO:0001882	Molecular function: nucleoside binding	Control	4.45	0.0002
GO:0031345	Biological process: negative regulation of cell projection organization	Control	71.15	0.0004
GO:0010171	Biological process: body morphogenesis	Control	67.59	0.0004
GO:0007492	Biological process: endoderm development	Control	46.61	0.0008
GO:0021954	Biological process: central nervous system neuron development	Control	42.24	0.001
GO:0050768	Biological process: negative regulation of neurogenesis	Control	39.76	0.0011
GO:0010721	Biological process: negative regulation of cell development	Control	37.55	0.0013
GO:0021953	Biological process: central nervous system neuron differentiation	Control	32.97	0.0017
GO:0030027	Cellular component: lamellipodium	Control	26.13	0.0026
GO:0031252	Cellular component: cell leading edge	Control	15.19	0.0076
GO:0044428	Cellular component: nuclear part	Control	3.16	0.0353
GO:0042995	Cellular component: cell projection	Control	3.99	0.0379
GO:0031981	Cellular component: nuclear lumen	Control	3.91	0.0399
GO:0005667	Cellular component: transcription factor complex	Control	6.25	0.0403

Summary of GO enrichment analysis of genes differentially acetylated for H4K5 in FC over control (FC) and control over FC (Control) by MACS and using Webgestalt GO analysis.

* Sorted according to treatment followed by P-value, using Benjamini-Hochberg correction for multiple comparisons.

**Table 2:** KEGG pathways enriched in MACS-identified genes differentially acetylated for H4K5 in FC and control in hippocampus

**Pathway**	**Treatment**	**Enrichment ratio**	*P*-value*
O-Glycan biosynthesis	FC	64.22	0.0005
Calcium signaling pathway	FC	13.29	0.0015
mTOR signaling pathway	FC	32.11	0.0018
Acute myeloid leukemia	FC	29.48	0.0021
Alzheimer's disease	FC	10.18	0.0033
Chronic myeloid leukemia	FC	21.16	0.0041
Cardiac muscle contraction	FC	20.43	0.0044
ErbB signaling pathway	FC	19.34	0.0049
Fc gamma R-mediated phagocytosis	FC	17.13	0.0062
Metabolic pathways	FC	3.66	0.012
Axon guidance	Control	32.99	0.0001
Calcium signaling pathway	Control	22.59	0.0003
Regulation of actin cytoskeleton	Control	20.2	0.0005
Long-term depression	Control	39.19	0.0012
Gap junction	Control	32.52	0.0018
Small cell lung cancer	Control	32.52	0.0018
GnRH signaling pathway	Control	29.68	0.0021
Melanogenesis	Control	28.84	0.0022
Vascular smooth muscle contraction	Control	22.99	0.0035
Focal adhesion	Control	14.77	0.0082

Summary of KEGG pathway enrichment analysis of genes differentially acetylated for H4K5 in FC over control (FC) and control over FC (Control) by MACS using Webgestalt KEGG analysis.

* Sorted according to treatment followed by P-value, using Benjamini-Hochberg correction for multiple comparisons.

In contrast, the 47 genes differentially acetylated for H4K5 in controls were classified into brain processes such as negative regulation of axogenesis, of neurogenesis, and of cell development, but also contributed to normal brain development and neuronal differentiation (Table 1; Additional file 1: Table S3 and 5). Pathway analysis for genes identified in controls showed enrichment for normal neuronal processes such as axon guidance, but also for genes associated with long-term depression, a form of synaptic plasticity typically associated with synaptic weakening (Table 2). The repressive functional categories and pathways enriched in controls suggest that training counteracts these pathways for memory formation. Alternatively, pathways upregulated in controls may be those that are needed to maintain homeostatic processes and basal neuronal functions in the absence of learning.

To validate whether genes differentially acetylated for H4K5 are also differentially expressed, we quantified mRNA expression of twelve randomly chosen genes called by MACS. mRNA levels were measured in hippocampal samples collected from animals from an independent CFC experiment to avoid sample or experimental bias associated with the ChIP-Seq. Seven out of twelve genes had significantly higher expression after CFC than in controls (Table 3). In contrast, in the cerebellum, a brain region not recruited for the formation of contextual fear memory, gene expression did not change after CFC, except for one (Table 3). Taken together, our data suggests that genes differentially acetylated for H4K5 are specific to memory formation in the hippocampus with CFC.

**Table 3:** Real-time quantitative PCR validation of MACS-identified genes differentially acetylated for H4K5 in FC over control in the hippocampus [HIP] and cerebellum [CER]

**Gene ID**	**Gene name**	Fold-change [HIP]*	**Significance [HIP]**	**Fold-change [CER]**	**Significance [CER]**
*Fbxl11*	F-box and leucine-rich repeat protein 11 [Lysine (K)-specific demethylase 2A (Kdm2a)]	4.31±1.40	0.05	1.04±0.05	NS
*Ryr3*	Ryanodine receptor 3	3.92±1.70	0.1	1.10±0.51	NS
*Akap6*	A kinase (PRKA) anchor protein 6	3.77±0.71	0.01	3.94±1.37	0.05
*Atp8a1*	ATPase, aminophospholipid transporter (APLT), class I, type 8A, member 1	3.35±0.46	0.01	0.96±0.23	NS
*Nrxn1*	Neurexin I	2.99±0.29	0.001	1.21±0.58	NS
*Rnf220*	Ring finger protein 220	2.98±1.84	NS	1.30±0.53	NS
*Kcnd2*	Potassium voltage-gated channel, Shal-related family, member 2	2.94±0.36	0.001	0.46±0.11	NS
*Grid1*	Glutamate receptor, ionotropic, delta 1	2.92±0.60	0.05	1.15±0.33	NS
*Col2a1*	Collagen, type II, alpha 1	2.35±0.42	0.01	1.01±0.21	NS
*Serpina9*	serpin peptidase inhibitor, clade A (alpha-1 antiproteinase, antitrypsin), member 9	1.90±0.90	NS	0.82±0.40	NS
*Megf11*	Multiple EGF-like-domains 11	1.71±0.67	NS	1.01±0.33	NS
*Ntrk2*	Neurotrophic tyrosine kinase, receptor, type 2	1.09±0.15	NS	1.32±0.20	NS

* Ranked according to fold-change in HIP.

Shown as fold-change over control ± SEM.

*NS* not significant.

## Discussion

The present study provides a comprehensive genome-wide analysis of H4K5ac in the hippocampus following fear memory formation, and identifies a novel set of genes associated with H4K5ac induced by learning. It demonstrates that H4K5ac is a ubiquitous histone PTM in the genome, present on one-third of genes with above average H4K5ac in the adult mouse hippocampus. Genes associated with high H4K5ac, in both promoter and CDS, are highly expressed, but H4K5ac is most prominent within 1000 kb upstream of the TSS. Our results suggest that H4K5ac may be required in both the promoter and CDS, over the entire length of the gene, for transcription of full and intermediate transcripts and that the presence of H4K5ac is a reliable marker of actively transcribed genes. However, we found that enrichment of H4K5ac in the promoter is determined, to an extent, by TF binding in which the absence of distal TFBS, 150 bp upstream of the TSS, dramatically increases H4K5ac enrichment in the promoter. We also provide evidence that H4K5ac may be a hallmark of activity-dependent genes that are expressed with learning. By identifying genes differentially acetylated for H4K5, we have uncovered key genes, both known and novel, involved in memory formation. These genes are specific to functions and pathways involved in synaptic plasticity and memory formation, but also to basic cellular processes, with learning.

The finding that promoters of ~80% of genes are acetylated above average for H4K5 regardless of training and that, of those, two-thirds are also acetylated for H4K12, is consistent with studies of other histone PTMs. In human cell lines, for instance, the promoters of 70% of genes were enriched for both H3K9ac and H3K14ac, of which >95% were also enriched for H3K4me3 [42]. It suggests that histone PTMs are ubiquitous in the genome, but it raises the question of whether their specificity depends on a few dominant modifications or a combination of histone PTMs, the extent to which multiple nucleosomes are modified in succession, and whether positioning of modified nucleosomes is a factor [26,50]. We found that ~15% of genes with above average H4K5ac are unique to FC and that genes differentially acetylated for H4K5 with learning are conducive to memory formation. This suggests that approximately 1000 out of 20,000 known protein-coding genes, or 5% of all genes, may be associated with memory in the hippocampus. At the moment, it is unclear what percent of genes are actively transcribed with learning, but synaptic proteins alone number 7,000, of which the postsynaptic density comprises more than 1000 proteins [51-54].

**Figure 6:**
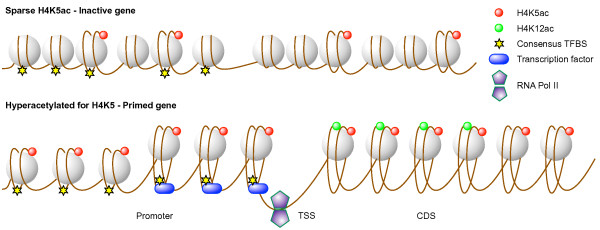
**Model depicting gene priming through hyperacetylation of H4K5.** Model depicting the potential role of H4K5ac preceding gene transcription. Sparse acetylation of H4K5 on nucleosomes associated with the promoter region is not sufficient for the recruitment of TFs necessary to initiate transcription. In contrast, hyperacetylation of H4K5 on nucleosomes along the promoter opens chromatin to allow access to large stretches of DNA for the recruitment of TFs proximal to the TSS. Upon binding, TFs may temporarily displace nucleosomes carrying the H4K5ac mark. In the CDS, H4K5ac and H4K12ac proximal to the TSS are necessary for transcriptional elongation by RNA polymerase. H4K5ac may be required throughout the gene body for full extension and/or transcription of splice variants such as *Phactr3*, whereas H4K12ac may only be needed in the CDS proximal to the TSS. This model proposes that hyperacetylation of H4K5 is needed at the promoter to prime genes for transcriptional initiation but also in the CDS for transcriptional elongation.

Differential acetylation analysis suggests that learning may target memory-specific genes for hyperacetylation over those normally acetylated for H4K5 under control conditions. Our data also show that H4K5ac is a reliable predictor of actively transcribed genes and that its level of enrichment correlates with the level of gene expression. Based on these observations, we propose that the prevalence of H4K5ac in the promoter may be a means to prime specific genes to facilitate their expression upon training or practice for rapid stabilization of the memory trace (Figure 6). Although mature neurons and glia are fully differentiated, our notion of priming is reminiscent of gene bookmarking in mitotic cells, whereby cells retain a ‘memory’ for patterns of gene expression through DNA and histone modifications following exit from mitosis [26,55,56]. Such a priming mechanism would be advantageous for the rapid induction of memory-specific genes following learning. However, it is currently not known how nucleosomes are positioned and modified with transcriptional activity or subsequent activity over time – whether they are depleted, displaced, or their modifications altered to retain a trace of prior activity [57-60].

Consistent with the notion of priming genes with repeated learning, approximately half of the genes we identified by peak-calling are involved in cognitive processes, while the other half has not been previously associated with memory processes. For instance, *Phactr3* (phosphatase and actin regulator 3), also known as *Scapinin* (scaffold-associated PP1-inhibiting proteins), is an interesting candidate with respect to memory as it is transcribed primarily in the brain and in tumors but has been relatively unstudied in the context of memory [40,61]. Likewise, *Pik3cd*, involved in the immune response and in cancer is implicated in the mTOR pathway with *Ddit4* (also known as *Redd1*) and *Tsc1/2*. Recent studies have linked *Tsc1/2* dysregulation to cognitive deficits associated with tuberous sclerosis and identified this gene as a potential target to treat autism [62,63]. *Ddit4* has also been implicated in Alzheimer’s disease and is therefore highly relevant for memory processes [64,65].

A notable feature of our findings is the considerably large number of intergenic loci found to carry H4K5ac. Our observation that genic regions only accounted for one-quarter of the 20,238 peaks differentially acetylated for H4K5 suggests that, in addition to gene bodies, H4K5ac is highly interspersed throughout intergenic regions. These regions are thought to give rise to noncoding RNAs or microRNAs that may potentially regulate genes. Indeed, the differentially acetylated targets we identified through both peak-calling algorithms and criteria-based selection methods included many known and novel noncoding RNAs. The recent discovery by the ENCODE consortium of an additional 30,000 intergenic and antisense TSS in the genome suggests that previously defined limits of what constituted genic regions, and gene annotations we used in this study, were incomplete and underestimated the activity of these novel intergenic regions [66]. Additionally, the ENCODE finding that nearly three-quarters of the genome can be transcribed at any given time, whether in genic or intergenic regions, suggests that the ubiquity of H4K5ac is to be expected if, as in our study, H4K5ac is a modification associated with active transcription and is required to transcribe intergenic regions [66].

Finally, another important question raised by our study is whether histone PTMs participate in the recruitment of transcriptional machinery. Although low intrinsic nucleosome occupancy has been documented in promoter regulatory regions, TFBS, and origins of replication in yeast [67,68], p53 was found to preferentially bind DNA sites strongly associated with nucleosomes over sites with relatively low nucleosome occupancy [69]. Our data show that actively transcribed genes with a conserved TFBS in positions proximal to the TSS have increased enrichment for H4K5ac in the promoter. Similarly, the ENCODE studies have shown that particular sets of TFs are strongly associated to proximal promoter regions and that the spatial positioning and structural motif of TFBS in these regions is highly conserved across many human cell lines [43,44]. This may suggest that nucleosomes demarcate positions of accessibility proximal to the TSS and, with appropriate modifications, open consensus sites to allow TF recruitment and binding. Other studies have shown that H3K9ac and H3K14ac are critical for the recruitment of TFIID in the promoter to initiate transcription [42,70]. Once bound, however, it is not yet known whether nucleosomes are deacetylated or evicted from the promoter of actively transcribed genes.

## Conclusion

Our study newly suggests that H4K5ac is induced in an activity-dependent manner in the adult mouse hippocampus where it may prime genes for rapid expression following repetitive learning. We propose that hyperacetylation of H4K5 proximal to the TSS in the promoter facilitates the recruitment of TFs and is associated with rapid gene expression following reinforced learning (Figure 6) [71]. Many questions still remain about chromatin remodeling and the extent to which it regulates gene expression in biological functions. However, this study provides new insight into chromatin remodeling in cognitive processes in a manner that is unbiased and independent of predefined genetic associations. Complementary genome-wide studies will be required in the future to comprehensively map the ensemble of histone modifications regulating genetic programs in cognitive and other biological processes.

## Methods

### Animals and contextual fear conditioning

Experiments were conducted using adult C57Bl6/J males (4–7 months old). Mice were housed under standard conditions with a 12 hour reversed light–dark cycle and access to food and water *ad libitum*. All animals were maintained in accordance with the Federation of Swiss Cantonal Veterinary Office and European Community Council Directive (86/609/EEC) guidelines.

Mice were habituated to the testing room and handled for three days prior to training and testing. They were then trained in a contextual fear-conditioning paradigm using a TSE Fear Conditioning System. The training consisted of a 3 min. exposure to the conditioning context followed by a brief electric shock (0.7 mA for 1s), then left for an additional 3 min. in the conditioning context. Mice that were not re-conditioned were euthanized 1 hour after the initial fear-conditioning session. Mice that were to be further fear-conditioned were trained on the second day and the memory test performed 24 hours later on the third day. Single trial CFC is known to produce a robust, long-lasting memory, however subsequent training has been shown to strengthen the memory and prevent random association of shock with re-exposure [6-10]. Furthermore, as re-exposure to the context on day 3 increased freezing, euthanasia was performed within one hour of the memory test on day 3, but before the 6-hour reconsolidation window and before extinction could take place [3,29]. The control group was handled and trained in the same manner but without a foot shock.

Comparisons between groups were analyzed by paired student’s t-test or one-way ANOVA with Tukey *post hoc* analysis, where appropriate. GraphPad Prism was used for statistical analysis and significance was set at *p ≤ 0.05, **p ≤ 0.01, and ***p ≤ 0.001. All data are shown as mean ± SEM.

### Nuclear extraction and Western blots

Nuclear protein extraction was performed as previously described with the following modifications [13]. Hippocampi were dissected and homogenized in 100 μl nuclear inhibition buffer (NIB) at pH 7.4 containing 3.75 mM Tris–HCl, 15mM KCl, 3.75 mM NaCl, 250 μM EDTA, 50 μM EGTA, 30% (v/v) glycerol, and 15 mM β-mercaptoethanol, with the addition of 1:200 proteinase inhibitor cocktail (Sigma-Aldrich), 1:500 PMSF (Sigma-Aldrich) and 1:100 phosphatase inhibitor cocktail (Sigma-Aldrich). The structures were then uniformly homogenized with a 22G syringe and centrifuged at 14,000 rpm for 30 min. The supernatant and pellet, containing cytoplasmic and nuclear material, respectively, was separated and resuspended in another 100 μl NIB with appropriate inhibitors. The pellet was re-homogenized with a 26G syringe and centrifuged at 14,000 rpm for 30 min.

15 μg of proteins from nuclear extracts was mixed with 4× LDS sample buffer (NuPage; Invitrogen) and 10% β-Mercaptoethanol to a final volume of 20 μl and loaded on a Novex 4-12% Bis-Tris Gel (NuPage; Invitrogen). Proteins were then transferred onto a nitrocellulose membrane (Bio-Rad), blocked (Rockland IR blocking buffer; Rockland Immunochemicals), and incubated with primary and secondary antibodies. Whole purified histones were run in parallel to confirm histone subunits (ImmunoVision) and Precision Plus protein dual color standards were used to determine molecular weights (Bio-Rad Laboratories). Bands were identified and quantified using an Odyssey IR scanner (LI-COR Biosciences) and the H4K5ac (11 kDa) signal was normalized to β-actin (42 kDa). Primary antibodies used were anti-acetyl H4K5 [72] (1:1000; Millipore) and monoclonal β-actin (1:1000; Sigma-Aldrich); secondary antibodies used were goat-anti-rabbit (IRDye 680 nm; 1:10,000) and goat-anti-mouse (IRDye 800 nm; 1:10,000; LI-COR Biosciences).

### Quantitative real-time PCR

Total RNA was extracted from hippocampus using TRIzol reagent and 1 μg of RNA was reverse-transcribed using the SuperScript First Strand Synthesis II system (Invitrogen). Equal amounts of cDNA from each sample were run in duplicate along with an endogenous control, *Gapdh*, on a Light Cycler 480 (Roche AG). Crossing point (Cp) values, which are more reliable and reproducible than Ct values, were obtained using the second derivative maximum method (Roche AG). Comparative analysis on Cp values was performed and expressed as fold change over the average of controls [73]. Mean and SEM values were obtained for each and analyzed using two-tailed paired t-tests to determine statistical significance (p <0.05). Oligonucleotides used for quantitative real-time PCR are listed in (Additional file 1: Table S6).

### Chromatin immunoprecipitation

Chromatin immunoprecipitation (ChIP) was performed as previously described [13], with the following modifications. Briefly, three hippocampal samples for each group were individually cross-linked with 1% formaldehyde, quenched with 0.125 M glycine, and spun down at 1500 rpm for 5 min at 4°C. To isolate chromatin, samples were washed and homogenized in 2 ml cell lysis buffer containing proteinase and phosphatase inhibitors with a Dounce homogenizer. Samples were centrifuged at 4000 rpm for 5 min. and homogenized again in 1 ml nuclear lysis buffer with inhibitors. DNA was sheared using a Baendelin Sono Plus to a fragment length of 600–800 bp. Total genomic DNA (input) was quantified and 80 μg of chromatin from each sample was immunoprecipitated overnight at 4°C with either 5 μl of anti-acetyl-H4K5 (07–327; Millipore) or 5 μl of IgG (17–685; Millipore) as a negative control. After incubation, nucleosome complexes were isolated with 60 μl of protein A agarose/salmon sperm DNA slurry (Millipore) for 1 h at 4°C. The complexes were washed and dissociated from the beads by incubation in 1% SDS in TE and nuclear lysis buffer at 65°C for 10 min. Histones were then digested with proteinase K for 1 h at 45°C and the DNA was finally extracted with phenol/chloroform/isoamyl alcohol and ethanol precipitation. DNA concentrations were measured on a Nanodrop (Thermo Fisher Scientific) and further verified on a Qubit fluorometer (Invitrogen). Uniformity of fragment size and quality control was validated on a 2100 BioAnalyzer (Agilent Technologies).

### ChIP-Seq library preparation

Library preparation was according to recommended guidelines (Life Technologies). From both ChIP and input control samples, 200 ng of DNA was further sonicated at 4°C to a mean fragment size of between 100 to 150 bp using the Covaris S2 sonicator. The DNA was then end-repaired using end-polishing enzymes such that damaged DNA with protruding 5’ or 3’ ends were blunt-ended and phosphorylated. Following repair, the samples were purified using a column purification kit and the blunt ends were ligated with 1 μl of multiplex adaptors. The ligated samples were then nick translated and amplified according to the SOLiD Fragment Library Barcoding protocol and column purified separately. The libraries were then quantitated using a Qubit fluorometer. 20 μl of each library was size-selected for ligation products of 170–230 bp using 2% E-gels and pooled following gel purification. Finally, equimolar amounts of each barcoded library were mixed together before ePCR followed by sequencing.

### SOLiD sequencing and mapping statistics

Sequencing was performed on an Applied Biosystems SOLiD 3 platform. Image acquisition and base calling was automated on the SOLiD Instrument Control Software system. The color space reads were mapped and aligned to the current assembly of the mouse genome (mm9, NCBI Build 37 dating July 2007; UCSC genome browser) using the mapping tool of the Bioscope v1.2.1 software suite (Applied Biosystems). Only reads with a maximum of 4 failed color calls and quality values larger than 8 were considered for contiguous mapping. The reads were mapped allowing a maximum of 6 color mismatches and reads with up to 10 mappings on the genome were reported in a SAM file. This file was used for subsequent identification of enriched regions. Sequence data from this study has been submitted to NCBI Gene Expression Omnibus database (http://www.ncbi.nlm.nih.gov/geo/) and assigned the identifier (accession no. GSE30325).

From a total of 309 million (309,346,614) 50-bp ChIP-seq reads, 230 million (229,838,436; 74.3%) were uniquely mapped to the current mouse reference genome with a mismatch allowance of ≤ 6 per 50 consecutive bases (Additional file 1: Table S1). The total number of sequenced reads was equivalent to ~6.2 complete mouse genomes (15.5 Gb), while the mappable reads were equivalent to ~4.6 genomes (11.5 Gb). We obtained an average of ~45 reads per promoter region, 783 and 894 reads per CDS for FC and control, respectively, with lower read counts for mock IgG-immunoprecipitated (IgG-IP) control samples (12 and 10 reads per promoter, 287 and 245 reads per CDS for FC and control, respectively) (Additional file 1: Table S1). An equivalent H4K12ac ChIP-seq dataset from Peleg et al. was obtained from Galaxy-Central (sm1186088) at <main.g2.bx.psu.edu/u/fischerlab/h/sm1186088> and re-analyzed using our workflow. With the H4K12ac dataset, we obtained 5.53 million total reads, of which 4.04 million were unique reads (73.1%) with an average coverage of 8.7 reads per promoter and 123 reads per CDS (Additional file 1: Table S1). The higher sequence coverage of H4K5ac in control, ~13.3% more mapped reads (4.9 million reads) compared to FC, may account for the larger number of genes identified in control with our exclusion criteria (>50 reads). The lower coverage (5.53 million reads in total) in H4K12ac may also explain the smaller percentage of genes found to overlap with H4K5ac.

### Differential peak calling and data mining analysis

Peak finding was performed using a Model-based Analysis of ChIP-Seq (MACS, version 1.3.7.1 - Oktoberfest) algorithm [46]. To determine genes differentially enriched for H4K5ac in the respective groups, we ran MACS on fear-conditioned against non-fear-conditioned control and vice versa. H4K5ac peaks were identified in MACS with the following parameters: effective genome size = 1.87e+09, tag size = 50, bandwidth = 300, m-fold = 4, and P-value cutoff = 1.00e-5. We also used the Statistical model for the Identification of chip-Enriched Regions (SICER, version 1.1) to call differentially acetylated peaks between groups [47]. We used the following parameters for SICER: redundancy threshold = 1, window size = 200, fragment size = 150, effective genome fraction = 0.7, gap size = 400, FDR = 1.00e-3, and filtered post-analysis for genes with P-value = 1.00e-5. We further compared results to the Genomatix NGS analyzer with Auto-Claverie algorithm with the following parameters: window size = 100 and P-value = 0.05, filtered post-analysis for genes with P-value = 1.00e-5 [49]. EpiChip analysis was performed according to standard protocols (epichip.sourceforge.net), except gene scoring was performed ± 5000 from the 5’ start position [48]. H4K12ac ChIP-Seq data, by CFC in young mice, was obtained from the public repository at Galaxy-Central (sm1186088 at <main.g2.bx.psu.edu/u/fischerlab/h/sm1186088>). Control ChIP-Seq data for H4K12ac, for sample or experimental condition, was not available [4].

### Gene ontology and pathway analysis

To determine functional gene enrichment and interaction networks of genes differentially acetylated in fear-conditioned compared to non-fear-conditioned controls, we used the genes identified in MACS for functional annotation. From the 241 differentially acetylated regions identified in fear-conditioned over control, 115 unique peaks were associated in the promoter or coding region of genes. From the 77 differentially acetylated regions identified in control over fear-conditioned, 42 unique peaks were associated with gene bodies. We used The Database for Annotation, Visualization and Integrated Discovery (DAVID, v6.7; http://david.abcc.ncifcrf.gov) for the analysis of functionally-enriched genes in our respective gene lists [74]. Settings were set at a count threshold of 2 and EASE score of 0.1, a more conservative test than Fisher’s Exact test. We also used Web-based Gene Set Analysis Toolkit V2 (WebGestalt; http://bioinfo.vanderbilt.edu/webgestalt) for the analysis of functionally-enriched genes in our respective gene lists [75]. Genes were analyzed using a hypergeometric test with multiple adjustment using the method of Benjamini & Hochberg and categorized into their respective classes or pathway associations based on the Kyoto Encyclopedia of Genes and Genomes (KEGG; http://www.genome.jp/kegg/kegg2.html).

### Gene expression analysis

Gene expression data was obtained from the Gene Expression Omnibus repository at NCBI (http://www.ncbi.nlm.nih.gov/geo/query/acc.cgi?acc=GSE20270), and processed and analyzed with R/Bioconductor. Spot intensities were normalized using quantile normalization. For the comparison with the acetylation levels, the genes were subdivided in ten equally sized bins according to the average expression.

### Transcription factor binding site analysis

Known transcription factor binding sites (TFBS) were downloaded from the CisRED database [76]. A total of 223,000 binding sites was used to analyze whether the presence of a known TFBS at a given position in the promoter determines the acetylation level at that position. Genes were divided into expressed and unexpressed genes, and expressed genes were further subdivided into two groups depending on whether a TFBS was annotated at that position. For each group we computed the percentage of genes acetylated at position × in step widths of 10, from 0 to 2000 bp upstream of the TSS.

### Acetylation profile clustering

We computed acetylation profiles in the ± 2 kb region around the transcription start site (TSS) and used k-means clustering to subdivide the profiles into 5 clusters. We subsequently built cross-tabulation tables to check whether cluster membership correlates with the expression level and/or with the presence of a known TFBS in certain regions. Clusters were generated in an unsupervised fashion and correlation between acetylation scores and gene expression was computed using Spearman's rank correlation.

## Abbreviations

CDS: Coding sequence; CFC: Contextual fear conditioning; ChIP-Seq: Chromatin immunoprecipitation followed by sequencing; ChIP: Chromatin immunoprecipitation; Cp: Crossing point; DAVID: The database for annotation, visualization and integrated discovery; FC: Fear conditioning; FDR: False discovery rate; GO: Gene ontology; H2BK5ac: Histone H2B lysine 5 acetylation; H2BK12ac: Histone H2B lysine 12 acetylation; H2BK15ac: Histone H2B lysine 15 acetylation; H2BK20ac: Histone H2B lysine20 acetylation; H3K14ac: Histone H3 lysine 14 acetylation; H3K9ac: Histone H3 lysine 9 acetylation; H4K12ac: Histone H4 lysine 12 acetylation; H4K5ac: Histone H4 lysine 5 acetylation; H4K8ac: Histone H4 lysine 8 acetylation; HDAC: Histone deacetylases; IgG-IP: IgG-immunoprecipitated; KEGG: Kyoto encyclopedia of genes and genomes; MACS: Model-based analysis of ChIP-Seq; PCR: Polymerase chain reaction; PP1: Protein phosphatase 1; PTM: Posttranslational modification; SAHA: Suberoylanilide hydroxamic acid; SICER: Statistical model for the identification of chip-enriched regions; TF: Transcription factor; TFBS: Transcription factor binding site; TSS: Transcription start site; TTS: Transcription termination site; UTR: Untranslated region; WebGestalt: Web-based gene set analysis toolkit V2.

## Competing interests

The authors declare they have no competing interests.

## Authors’ contributions

CSP initiated and designed the study. CSP performed and/or oversaw fear conditioning experiments, ChIP for sequencing, peak-finding analysis, raw data analysis, GO analysis, RT-PCR validations, and wrote the manuscript. HR provided analysis of mapped reads, sequence read annotation and classification, gene expression correlation, and transcription factor binding site analysis. IMM provided interpretation of the data and critical revision of the manuscript. All authors have read and approved the manuscript.

## Supplementary Material

Additional file 1 **Supplementary figures and tables.** This file contains Supplementary Figures 1 to 5 and Supplementary Tables 1 to 6. Click here for file
