# Supplementary material for: Genome-wide analysis of H4K5 acetylation associated with fear memory in mice
Source: BMC Genomics. 2013 Aug 8;14:539. doi: 10.1186/1471-2164-14-539 (PMC3751108; doi:10.1186/1471-2164-14-539)
Supplement: Additional file 1 — Supplementary figures and tables. This file contains Supplementary Figures 1 to 5 and Supplementary Tables 1 to 6. [file 1471-2164-14-539-S1.pdf]

Genome-Wide Analysis of H4K5 Acetylation Associated with Fear Memory in Mice

C. Sehwan Park[1]*, Hubert Rehrauer[2], Isabelle M. Mansuy[1]*

[1] Brain Research Institute, Medical Faculty of University of Zürich,
and Department of Health Science and Technology, ETH Zürich
[2] Functional Genomics Center of Zürich
University of Zürich and ETH Zürich
Winterthurerstrasse 190
8057 Zürich
Switzerland

***Corresponding authors**:

Isabelle M. Mansuy
mansuy@hifo.uzh.ch
C. Sehwan Park
cpark@list.mavt.ethz.ch

Brain Research Institute
University of Zürich
Winterthurerstrasse 190
CH-8057 Zürich
Switzerland
Phone +41 44 635 3360
Fax +41 44 635 3303

Supplementary Figures:

Supplementary Figure 1. Correlation of gene expression to mock IgG-IP reads.

Supplementary Figure 2. Correlation of H4K5ac clusters to gene expression in control.

Supplementary Figure 3. Correlation of genes by acetylation and gene expression in the promoter, CDS, and 3'-UTR.

Supplementary Figure 4. Effect of TFBS on H4K5ac and IgG-IP clusters in control.

Supplementary Figure 5. Identification of genes differentially acetylated for H4K5 with peak-calling algorithms.

Supplementary Tables:

Supplementary Table 1. Mapping statistics following alignment of ChIP-Seq reads.

Supplementary Table 2. MACS analysis of H4K5ac-enriched genomic regions in FC over control.

Supplementary Table 3. MACS analysis of H4K5ac-enriched genomic regions in control over FC.

Supplementary Table 4. DAVID analysis of genes differentially acetylated for H4K5 in FC over control as identified by MACS.

Supplementary Table 5. DAVID analysis of genes differentially acetylated for H4K5 in control over FC as identified by MACS.

Supplementary Table 6. Oligonucleotides used for quantitative RT-PCR.

Sequence data from this study has been submitted to NCBI Gene Expression Omnibus database (http://www.ncbi.nlm. nih.gov/geo/) and assigned the identifier (accession no. GSE30325).

Supplementary Figures

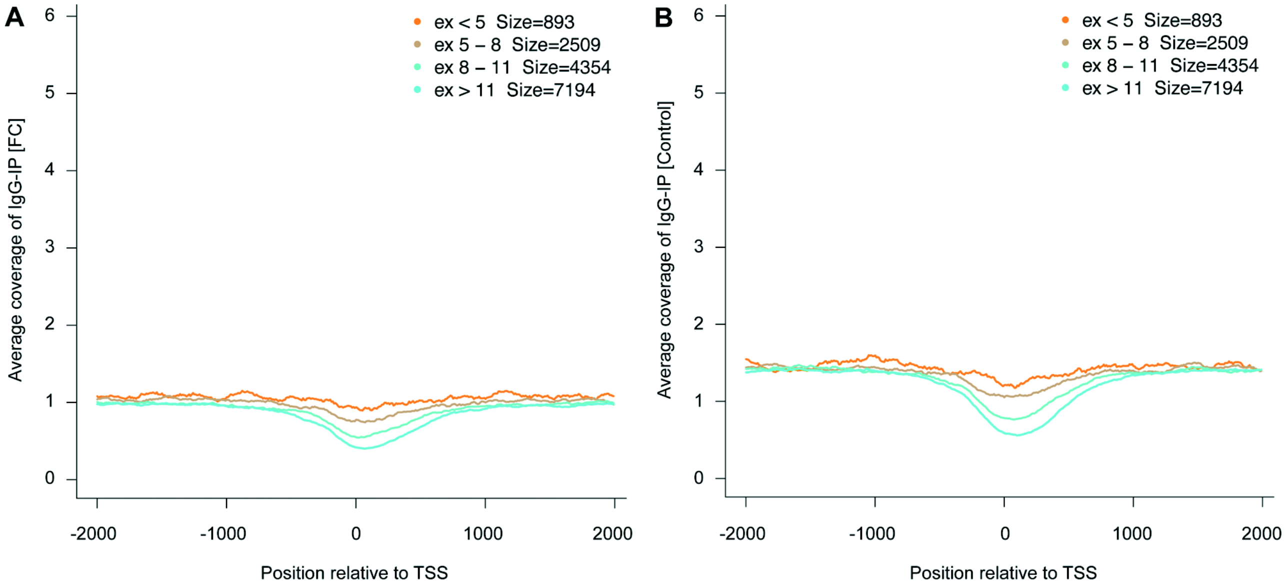

Supplementary Figure 1. Correlation of gene expression to mock IgG-IP reads. (A, B) Genes are categorized into four bins from lowest (ex < 5; orange) to highest (ex > 11; blue) expressed genes and correlated to average coverage for IgG-IP from (A) FC or (B) Control samples. Average read coverage ± 2 kb relative to the TSS.

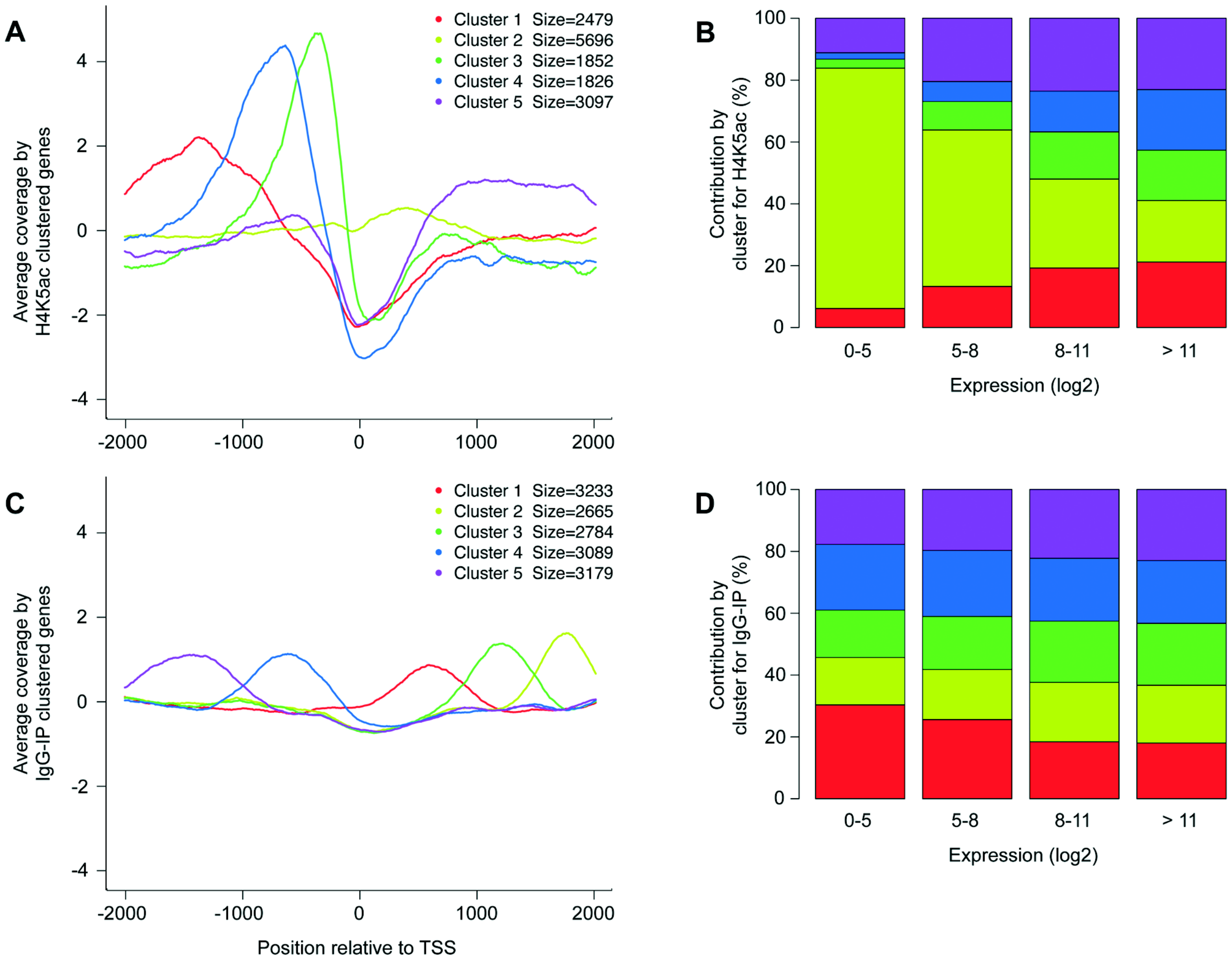

Supplementary Figure 2. Correlation of H4K5ac clusters to gene expression in control. (A) Genes acetylated for H4K5 in control are clustered based on acetylation profiles ±2 kb relative to the TSS. Clusters 1 (red), 3 (green), and 4 (blue) occur in promoter regions, cluster 5 (violet) occurs in the CDS, and cluster 2 (yellow) is unenriched for H4K5ac. (B) Contribution of gene clusters in control with respect to levels of gene expression show higher contribution of clusters enriched for H4K5ac with higher expression. (C) Gene clusters from IgG-IP reads in control are uniformly distributed and consist of clusters 4 (blue) and 5 (violet) in the promoter region, and clusters 1 (red), 2 (yellow), and 3 (green) in the CDS. (D) IgG-IP clusters show relatively no change in contribution with gene expression levels.

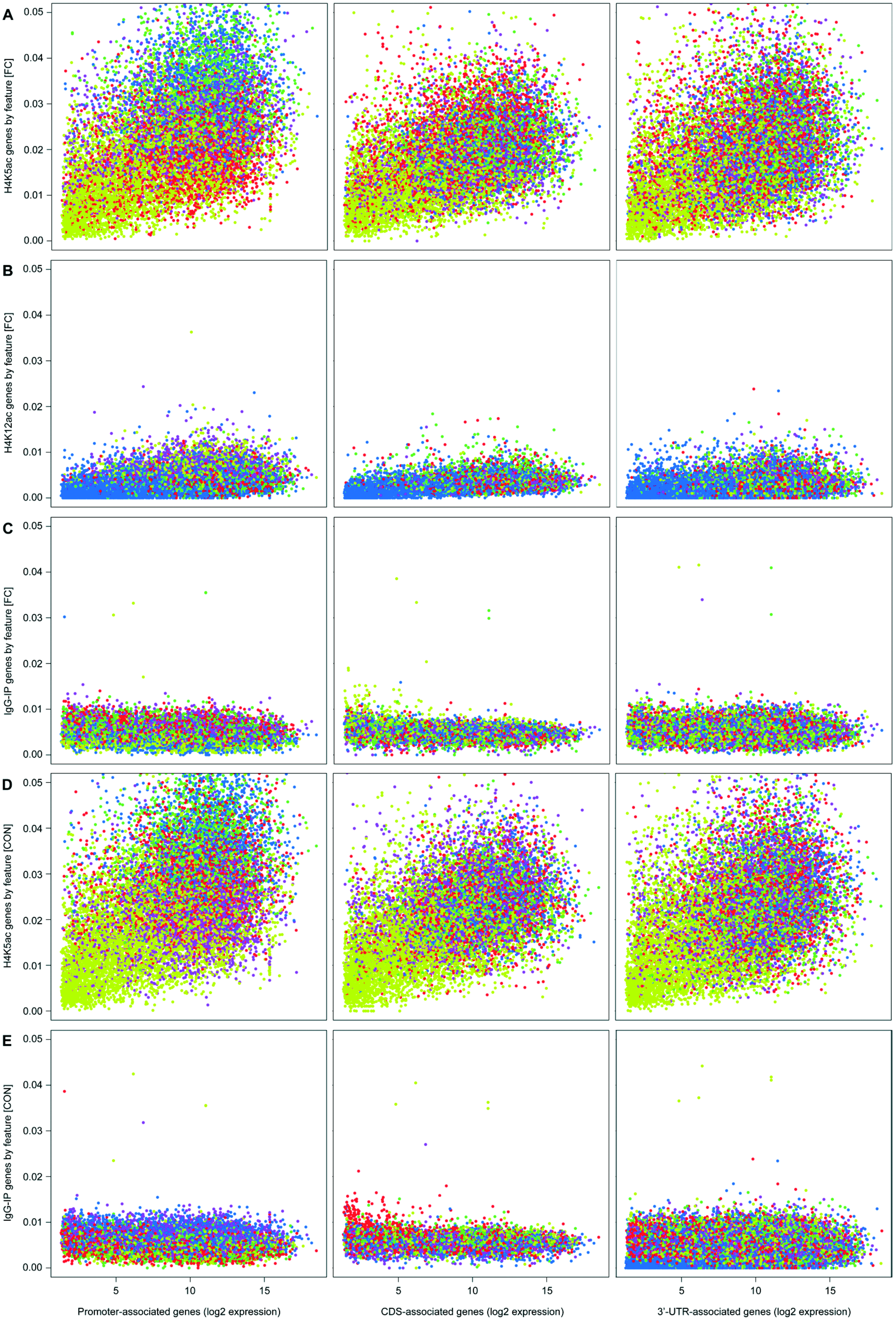
A
B
C
D
E
H4K5ac genes by feature [FC]
H4K12ac genes by feature [FC]
IgG-IP genes by feature [FC]
H4K5ac genes by feature [CON]
IgG-IP genes by feature [CON]
0.00
0.01
0.02
0.03
0.04
0.05
5
10
15
Promoter-associated genes (log2 expression)
CDS-associated genes (log2 expression)
3'-UTR-associated genes (log2 expression)

Supplementary Figure 3. Correlation of genes by acetylation and gene expression in the promoter, CDS, and 3'-UTR. (A) Correlation of gene expression to promoter-associated H4K5ac (left; r = 0.4624), CDS-associated H4K5ac (middle; r = 0.4586), and 3'-UTR-associated H4K5ac (right; r = 0.4058) by FC. (B) Correlation of gene expression to promoter-associated H4K12ac (left; 0.4040), CDS-associated H4K12ac (middle; r = 0.3899), and 3'-UTR-associated H4K12ac (right; r = 0.2679) by FC. (C) Correlation of gene expression to promoter-associated IgG-IP reads (left; r = -0.1352), CDS-associated IgG-IP reads (middle; r = -0.1424), and 3'-UTR-associated IgG-IP reads (right; r = -0.0750) by FC. (D) Correlation of gene expression to promoter-associated H4K5ac (left; r = 0.4681), CDS-associated H4K5ac (middle; r = 0.4883), and 3'-UTR-associated H4K5ac (right; r = 0.4222) in control (CON). (E) Correlation of gene expression to promoter-associated IgG-IP reads (left; r = -0.1053), CDS-associated IgG-IP reads (middle; r = -0.0990), and 3'-UTR-associated IgG-IP reads (right; r = -0.0413) in control (CON). Clusters are defined in Figure 4A, 4C, and 4E and Supplementary Figures 4A and 4C.

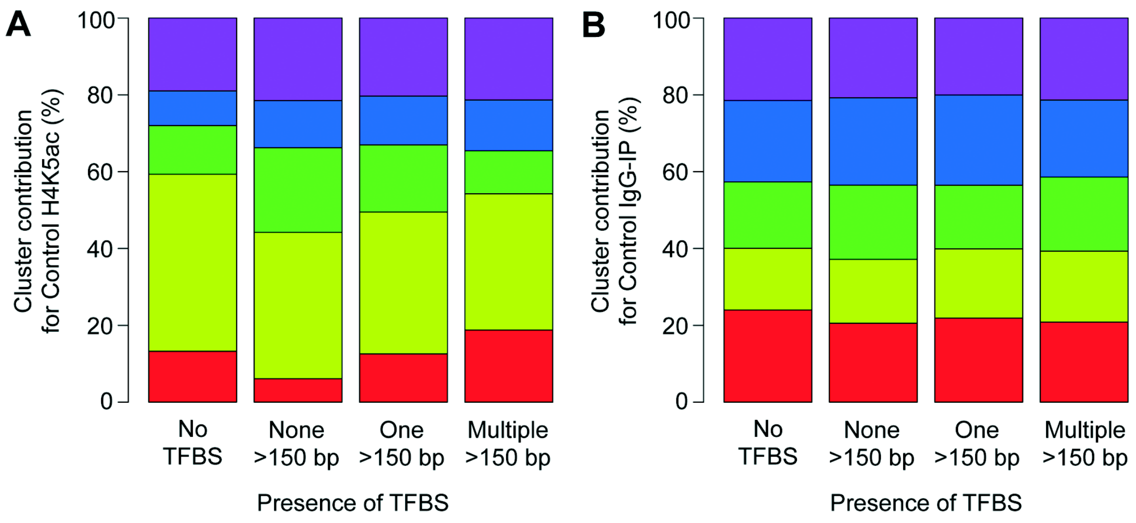

Supplementary Figure 4. Effect of TFBS on H4K5ac and IgG-IP clusters in control. The relative contribution of gene clusters for (A) H4K5ac and (B) IgG-IP in control with either no TFBS present in the promoter, or no TFBS, one TFBS, and multiple TFBS upstream of -150 bp relative to the TSS. Clusters are defined in Supplementary Figures 4A and 4C.

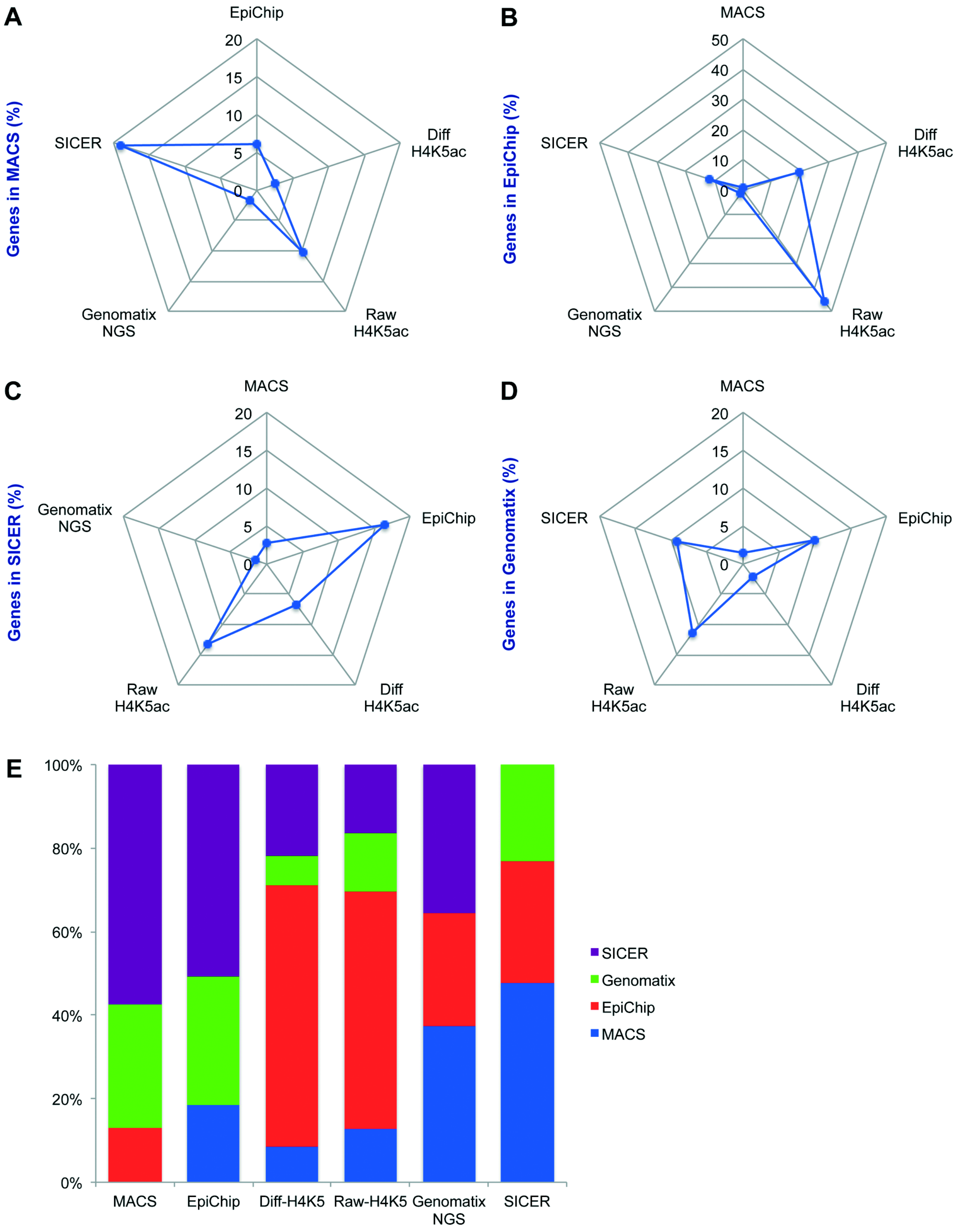
A
Genes in MACS (%)
EpiChip
20
15
10
5
0
SICER
Diff
H4K5ac
Genomatix
NGS
Raw
H4K5ac
B
Genes in EpiChip (%)
MACS
50
40
30
20
10
0
SICER
Diff
H4K5ac
Genomatix
NGS
Raw
H4K5ac
C
Genes in SICER (%)
MACS
20
15
10
5
0
Genomatix
NGS
EpiChip
Raw
H4K5ac
Diff
H4K5ac
D
Genes in Genomatix (%)
MACS
20
15
10
5
0
SICER
EpiChip
Raw
H4K5ac
Diff
H4K5ac
E
100%
80%
60%
40%
20%
0%
MACS
EpiChip
Diff-H4K5
Raw-H4K5
Genomatix
NGS
SICER
SICER
Genomatix
EpiChip
MACS

Supplementary Figure 5. Identification of genes differentially acetylated for H4K5 with peak-calling algorithms. Percentage of genes identified by (A) MACS, (B) EpiChIP, (C) SICER, and (D) Genomatix NGS against genes identified by other peak-calling algorithms, or against genes with tag densities with a threshold of greater than 50 reads in FC (Raw H4K5ac) or greater than 50 reads in FC and less than 40 reads in control in the promoter (Diff H4K5ac). Genes identified by MACS had the highest overlap with genes identified by SICER (19%), followed by Raw H4K5ac (10%) and EpiChip (6%). EpiChip had high overlap with Raw H4K5ac and Diff H4K5ac, with 46% and 19% of genes overlapping, respectively, followed by SICER at 12%. Since EpiChIP identified acetylated genes and determined the presence or absence of genes between two treatment groups, similar to a criteria-based approach, EpiChIP, Raw H4K5ac, and Diff H4K5ac identified a high percentage of the same genes. SICER had moderate overlap with EpiChip (16%), Raw H4K5ac (13%) and Diff H4K5ac (7%), and low overlap with MACS (2.7%) and Genomatix (1.6%). Genomatix had moderate overlap with Raw H4K5ac (11%), MACS (10%) and SICER (9%). (E) Proportion of genes identified by SICER (violet), Genomatix (green), EpiChIP (red), and MACS (blue) as a percentage of all genes identified by each algorithm and against Raw H4K5ac and Diff H4K5ac in a scaling matrix. The disproportionate number of genes found to overlap between algorithms can be attributed to the different number of genes identified by each algorithm, based on different parameters unique to each algorithm.

Supplementary Table 1. Mapping statistics following alignment of ChIP-Seq reads

Sample	Total reads	Reads mapped	Mapped [%]	No. of start points	Average reads [promoter]	Median reads [promoter]	Average reads [gene]	Median reads [gene]
**Control-H4K5**	54326574	41559675	76.5	36423222	46.16	45	894.22	280
**Control-IC**	60886714	47254187	77.6	37676866	27.78	28	644.23	219
**Control-IgG**	30808841	24126269	78.3	17969145	12.00	12	286.56	94
**FC-H4K5**	52343044	36651928	70.0	31310961	43.42	42	783.32	257
**FC-IC**	81416434	57848969	71.1	48795166	43.53	44	907.94	345
**FC-IgG**	29565007	22397408	75.8	15536790	10.16	10	244.71	79
**Sum Reads**	309346614	229838436	74.3					
**Young-H4K12**					8.68	7	122.73	46

Reads were mapped and aligned to the mouse reference genome for H4K5ac, input control (IC), and IgG-IP control (IgG-IP), for control mice and mice trained by FC. H4K12ac ChIP-Seq data was obtained from Galaxy-Central (sm1186088 at <main.g2.bx.psu.edu/u/fischerlab/h/sm1186088>), remapped and analyzed using our workflow.

Supplementary Table 2. MACS analysis of H4K5ac-enriched genomic regions in FC over control

Gene ID	Chromosome	Start	End	Length	Summit	Tags	- 10*log10 (*P*-value)	Fold enrichment	FDR (%)
1110012D08Rik	10	128239616	128240116	501	158	27	54.76	4.9	67.12
2310007F21Rik	9	63472539	63473691	1153	401	66	61.17	3.73	66.67
2310007H09Rik	7	139891162	139893999	2838	592	187	62.41	2.79	62.12
4930427A07Rik	12	114400852	114402134	1283	935	75	78.23	3.86	41.18
4933407N01Rik	11	30853345	30853979	635	302	34	66.76	4.6	65.91
Abcc1	16	14473228	14473822	595	287	28	50.97	4.85	73.52
Acot7	4	151595941	151596961	1021	281	57	57.53	3.58	61.68
Adcy5	16	35230851	35232327	1477	1351	96	70.39	3.29	58.06
Adcy5	16	35232534	35233843	1310	308	77	53.19	3.71	69.71
Akap6	12	54033421	54033824	404	294	19	54.36	5.3	65.16
Ank	15	27424248	27425770	1523	988	84	51.69	3.39	72.77
Ank3	10	69350962	69352047	1086	361	60	50.78	3.36	73.09
Ap3b1	13	95138353	95139054	702	438	39	53.31	3.62	69.94
App	16	85001974	85002944	971	352	48	55.92	3.62	63.64
Arhgap26	18	39345666	39347697	2032	1731	124	51.44	2.77	71.56
Arhgef3	14	28179583	28181218	1636	260	86	61.14	3.58	65.79
Atp8a1	5	68053560	68054175	616	338	32	57.06	6.26	60.87
Bace1	9	45661241	45662797	1557	1002	93	60.74	3.89	65.38
Bach2	4	32647574	32649648	2075	657	120	71.28	2.82	53.57
BC019943	8	32265962	32266413	452	183	20	52.57	5.84	72.28
BC033915	9	45862143	45863455	1313	261	84	53.7	3.07	68.67
Blmh	11	76758134	76759132	999	839	60	94.56	4.85	40
Bola3	6	83299564	83300416	853	196	53	63.89	4.55	61.82
Capn8	1	184539238	184539696	459	124	24	61.24	4.98	67.12
Chd7	4	8725428	8726522	1095	256	65	52.96	3.57	68.68
Chd9	8	93543490	93543979	490	202	26	55.6	4.74	64.44
Clcn4-2	7	7251425	7253416	1992	274	109	80.73	3.45	50
Clip3	7	31092747	31093807	1061	769	52	56.67	4.66	63.33
Col27a1	4	62936537	62937232	696	455	39	53.84	4.37	69.51
Col2a1	15	97822728	97823455	728	408	38	73.29	6.3	65
Cuedc1	11	87938994	87940398	1405	492	81	64.93	3.26	64.58
Ddit4	10	59419144	59422275	3132	2097	214	50.55	3.12	74.56
Dhdh	7	52730353	52731352	1000	323	53	63.15	4.06	66.67
Dnajc7	11	100481102	100481892	791	663	45	58.84	3.62	60
Dnm2	9	21245723	21246757	1035	719	64	50.47	3.23	74.46
Dtx1	5	121130423	121131227	805	232	45	53.17	4.08	69.32
Dusp26	8	32201922	32202863	942	581	53	58.33	4.53	61.62
Dym	18	75433509	75435656	2148	1841	115	56.72	3.39	63.03
Eftud1	7	89919723	89920732	1010	689	83	52.15	3.43	71.5
EG328231	13	41753894	41755114	1221	278	65	60.95	4.41	64.94
Erc2	14	28715423	28715912	490	173	29	57.79	5.14	60.58
Fam164c	12	86629532	86630194	663	248	34	50.88	3.88	73.42
Fbxl11	19	4334110	4334698	589	265	31	59.72	5.33	65.12
Frmd5	2	121378180	121378738	559	307	30	64.49	5.61	64
Galntl1	12	81626980	81630339	3360	1064	235	53.57	2.26	69.82

Glrx2	1	145585927	145586529	603	336	32	64.34	5.07	61.54
Glt25d2	1	154309550	154309947	398	261	19	51.96	5.53	73.1
Gnal	18	67292215	67292652	438	154	20	54.61	5.3	66.22
Gng3-Bscl2	19	8911425	8912681	1257	293	74	54.51	3.32	65.77
Gpd1	15	99554121	99555643	1523	609	98	57.81	3.8	61.17
Grid1	14	35680988	35681561	574	278	27	50.91	5.35	73.3
Homer1	13	94169627	94170274	648	188	36	50.06	3.32	74.17
Hyou1	9	44198915	44199731	817	127	37	57.7	3.89	60
Itsn1	16	91770169	91770925	757	357	45	61.19	3.43	67.57
Kcnd2	6	21246050	21246462	413	164	22	51.85	5.76	72.86
Kcnu1	8	27021784	27023191	1408	295	85	54.44	2.87	65.79
Lrp1b	2	42051732	42052154	423	219	19	51.48	5.84	72.6
Macf1	4	123241159	123243431	2273	1195	154	80.29	3.24	46.67
Mad1l1	5	140745960	140746713	754	319	38	56.37	4.33	61.42
Man1c1	4	134149132	134149866	735	460	41	55.39	4.04	65.94
Mapre3	5	31132547	31133186	640	369	30	56.95	5.9	61.21
Mboat2	12	25555975	25556753	779	194	44	50.12	3.96	74.37
Mdga2	12	68230792	68231486	695	140	41	56.65	3.28	62.81
Megf11	9	64441569	64442007	439	314	21	55.14	5.94	64.79
Mgll	6	88770740	88771927	1188	874	65	58.19	4.12	62
Mlxip	5	123866851	123867917	1067	709	68	71.04	3.71	55.17
Msra	14	64763834	64765925	2092	702	135	59.2	2.77	62.22
Mvp	7	134151638	134152815	1178	311	68	69.08	3.55	59.46
Mybphl	3	108175187	108176388	1202	245	68	67.36	3.61	64.29
Myo7a	7	105250599	105251841	1243	921	95	64.09	4.4	61.11
Ndufa13-Tssk6	8	72427295	72428236	942	325	55	57.26	3.99	60.91
Nfix	8	87289040	87290720	1681	290	99	53.06	2.39	69.44
Nrp2	1	62763181	62764730	1550	246	86	53.68	3.34	67.86
Nrxn1	17	90933664	90934143	480	181	26	50.45	4.35	74.25
Nrxn2	19	6450022	6451702	1681	287	112	72.84	3.04	56.52
Ntrk2	13	59111781	59112426	646	282	39	67.65	5.1	65
Nuak1	10	83856325	83859850	3526	1466	221	59.85	2.44	63.53
Pacrg	17	10633926	10635965	2040	741	130	73.87	3.91	63.16
Pgs1	11	117847065	117848200	1136	441	70	54.45	3.85	66.23
Phactr3	2	177978069	177979491	1423	615	89	53.99	2.93	68.32
Pik3cd	4	149048746	149049553	808	263	38	94.47	5.03	33.33
Plekha6	1	135164171	135165378	1208	825	69	55.18	3.27	64.54
Ppm1h	10	122228941	122229630	690	257	39	54.37	4.8	65.58
Prkcdbp	7	112628633	112629610	978	145	58	50.99	3.25	73.85
Pum1	4	130309674	130310586	913	324	48	54.31	4.27	65.38
Rab31	17	66120603	66121615	1013	789	56	65.27	3.96	65.22
Rgnef	13	98849079	98849700	622	244	36	57.14	5.62	59.29
Rnf220	4	117088161	117088767	607	292	29	51.46	6.3	71.9
Rpgr	X	9792467	9792935	469	305	24	59.6	5.43	64.37
Ryr3	2	112856455	112856985	531	313	26	59.15	6.72	61.54
Sap30bp	11	115815431	115816728	1298	419	77	50.14	2.93	74.68
Sema7a	9	57795278	57800233	4956	3000	325	68.82	2.58	60.53
Serpina9	12	105251442	105251882	441	221	21	55.2	5.97	65
Sfi1	11	3069263	3074906	5644	4226	549	56.39	2.53	61.9
Sfi2	11	3090863	3095267	4405	504	468	57.95	2.36	62.38
Sfrs16	7	20187428	20188264	837	337	47	54.3	3.91	65.61
Slc24a2	4	86788722	86789442	721	435	43	53.72	3.52	69.09

Slc43a2	11	75364821	75366785	1965	1075	133	50.25	2.73	74.58
Slc8a1	17	82035622	82036243	622	331	33	51.95	5.03	72.73
Slc9a1	4	132954499	132955715	1217	571	65	64.25	3.94	60.38
Slc9a9	9	95054767	95055701	935	459	46	50.94	3.33	73.64
Slco2b1	7	106816987	106818064	1078	507	57	51.98	2.85	73.47
Ssbp3	4	106594937	106596362	1426	266	76	60.21	3.84	61.9
St3gal2	8	113473923	113475265	1343	1074	71	69.77	4.15	55.88
St3gal4	9	34914959	34916615	1657	1290	99	52.46	3.29	71.12
Stat5b	11	100712263	100713084	822	464	44	51.24	3.62	73.11
Tbc1d16	11	119028647	119029122	476	174	24	51.78	4.13	73
Tm4sf5	11	70323360	70323958	599	440	32	50.57	3.83	74.45
Tmem63b	17	45800406	45801001	596	346	31	50.99	4.53	74.19
Tmod1	4	46107724	46109712	1989	618	123	72.69	3.86	58.33
Tpk1	6	43615181	43615996	816	613	55	52.97	3.26	69.06
Zc3h7b	15	81590622	81591235	614	270	34	55.09	5.9	64.34
Zfp551	7	13007286	13008100	815	500	46	51.98	3.68	73.33
Zhx2	15	57584648	57586251	1604	962	86	53.09	2.99	69.1
Zufsp	10	33669849	33670520	672	121	35	52.3	3.83	73.02
Mgat4a-Tsga10	1	37744815	37745607	793	591	42	51.58	3.75	73.53
Als2-Fzd7	1	59521532	59522143	612	267	30	53.54	5.03	69.41
Qsox1-Tor1aip1	1	157775720	157776146	427	255	19	50.42	5.26	73.62
Aldh8a1-Sgk1	10	21370476	21371498	1023	418	64	63.89	3.27	60.71
Cdc2l6-Slc22a16	10	40233516	40236200	2685	1979	188	70.78	2.85	60
Epyc-Atp2b1	10	97881022	97881759	738	168	34	59.24	4.74	62.92
Lemd3-Wif1	10	120417184	120417704	521	356	26	50.45	4.32	74.25
Tns3-Hus1	11	8857964	8858489	526	254	25	52.47	4.76	71.51
Arf1-Wnt3a	11	59041830	59042711	882	263	55	60.58	4.24	64.56
Nf1-Rab11fip4	11	79403641	79404371	731	124	42	56.46	3.92	63.93
OTTMUSG00000002038-OTTMUSG00000002043	11	94578096	94579223	1128	256	62	60.45	3.39	61.45
Myst2-5730593F17Rik	11	95186866	95188019	1154	299	55	71.62	4.04	51.85
Cacng4-Cacng5	11	107667036	107667466	431	157	20	55.67	6.37	64.18
Hs1bp3-Rhob	12	8408846	8409661	816	565	37	57.6	5.76	62.26
Hs1bp3-Rhob	12	8455474	8458840	3367	588	213	51.48	2.91	72.25
Stxbp6-Foxg1	12	46175587	46176414	828	266	45	73.01	5	59.09
Arhgap5-Akap6	12	53772582	53773052	471	225	21	50.09	4.92	74.48
Abcd4-7420416P09Rik	12	85958439	85959170	732	356	40	50.75	3.55	72.77
Kcnk13-Psmc1	12	101311702	101312431	730	356	41	62.46	3.83	63.08
Clmn-	12	106103352	106104713	1362	474	100	93.97	3.7	28.57
Gpr141-Elmo1	13	20225030	20226202	1173	937	57	56.44	3.5	63.41
E2f3-Mboat1	13	30226671	30227306	636	387	35	57.37	4.53	60.55
Cks2-Secisbp2	13	51746480	51747071	592	354	27	58.45	5.13	60.2
Trpc7-Spock1	13	57253240	57254079	840	216	42	52.51	4.06	71.89
Cd180-Sfrs12	13	103910509	103911148	640	213	36	54.4	3.75	65.36
Ddhd1-LOC218963	14	46278035	46279134	1100	327	66	102	5.46	33.33
Peli2-6720456H20Rik	14	48991430	48992090	661	125	32	52.62	3.54	72.13
Jph4-Dhrs2	14	55765131	55766636	1506	324	85	62.84	3.48	62.9
Mtmr6-Fam123a	14	60981392	60982488	1097	436	63	67.22	3.86	65.12
AW549877-Oxct1	15	3975344	3976558	1215	912	69	63.05	4.19	63.33
Wdr67-BC030396	15	57815665	57816331	667	414	34	56.26	4.9	62.79
Wnt7b-Ppara	15	85429695	85431089	1395	1208	76	56.4	3.66	62.4
Hdac10-Mapk12	15	88959059	88959895	837	538	58	62.26	4.01	61.76
Slc2a13-Lrrk2	15	91502696	91503576	881	497	46	63.07	4.39	64.41

Snx4-Zfp148	16	33343895	33344965	1071	757	59	53.31	3.73	69.54
Parp9-Kpna1	16	35981543	35982037	495	283	27	55.72	7.92	63.91
Gap43-Zbtb20	16	42396727	42397157	431	272	21	53.85	5.72	69.94
Hlcs-Ripply3	16	94523910	94524681	772	478	45	82.9	5.22	38.46
Mllt4-EG635895	17	14043715	14044164	450	279	23	61.32	5.72	68.06
Flywch2-Srrm2	17	23923545	23924398	854	275	46	53.9	5.43	69.75
Stk38-Sfrs3	17	29144975	29145705	731	544	47	51.51	3.92	72.95
Spats1-EG668319-Aars2	17	45615728	45616491	764	405	47	68.02	5.03	64.1
Daam2-Kif6	17	49728652	49729417	766	267	38	69.62	4.59	60
Tbc1d5-Satb1	17	51344526	51345175	650	458	36	52.41	3.23	71.81
Ddx11-1110012J17Rik	17	66570611	66571171	561	281	23	53.1	7.43	69.49
Ndc80-Fam179a	17	71876466	71877521	1056	254	64	55.46	3.35	64.96
Galnt1-2700062C07Rik	18	24468105	24468644	540	311	30	61.93	4.14	63.38
2010001M09Rik-5133400G04Rik	18	35814942	35815573	632	153	33	62.04	4.56	65.22
Csnk1a1-Il17b	18	61825784	61826604	821	250	46	56.78	3.87	62.71
1810005K13Rik-Pqlc1	18	80452167	80452776	610	236	33	53.69	4.02	68.26
Ccs-Ccdc87-Ctsf-Actn3	19	4854158	4855061	904	343	54	54.24	3.58	65.19
Patl1-Osbp	19	12039184	12039815	632	427	35	51.15	4.53	72.43
Tle4-Psat1	19	15876224	15877056	833	189	47	56.24	3.39	62.31
Gda-1110059E24Rik	19	21645136	21645649	514	315	30	61.93	4.65	63.38
Smarca2-Gm815	19	26920021	26920849	829	719	40	50.67	3.44	73.33
Ranbp6-Il33	19	29976298	29976887	590	374	33	58.78	4.8	59.38
Pkd2l1-Scd3	19	44267148	44267756	609	287	33	57.26	5.22	60.36
Add3-Mxi1	19	53355855	53357336	1482	316	84	53.99	3.06	68.75
4930506M07Rik-Vax1	19	59158586	59159006	421	223	19	51.78	7.43	72.64
Gpr158-Myo3a	2	21839555	21840599	1045	130	59	55.36	3.24	65.47
Rxra-Col5a1	2	27620115	27621046	932	432	53	86.34	4.49	40
Cstad-1700001O22Rik	2	30532674	30534049	1376	430	102	60.51	3.66	62.96
Syt13-Trp53i11	2	93018989	93020222	1234	309	67	60.47	3.89	62.2
Ttc17-Lrrc4c	2	96157595	96158705	1111	767	67	62.91	3.05	63.93
26000010E01Rik-Commd9	2	101709503	101709860	358	125	17	50.5	6.37	74.35
Ldlrad3-Trim44	2	102130579	102131615	1037	341	60	60.53	3.99	63.75
Sqrdl-Sema6d	2	124028301	124028805	505	204	27	52.19	4.42	71.88
Banf2-Snx5	2	144030246	144031093	848	296	44	62.5	4.49	64.06
Dtd1-1700010M22Rik	2	144622551	144623005	455	229	20	51.65	5.79	72.91
H13-Id1	2	152561402	152562035	634	260	32	53.07	5.43	68.72
Dhx35-Gm826	2	158755713	158756122	410	238	20	58.98	6.37	59.57
Cebpb-Ptpn1	2	167686139	167686879	741	471	45	62.58	3.68	63.49
Slc7a11-Ccrn4l	3	50851899	50852729	831	128	39	51.12	4.23	72.09
Crtc2-Dennd4b	3	90069815	90070753	939	331	48	69.43	4.99	58.33
Slc6a17-Ubl4b	3	107321005	107321518	514	216	32	52.25	4.07	72.63
Gstm6-Gstm3	3	107748321	107750133	1813	942	100	65.23	3.34	63.83
Slc35a1-1700003M02Rik	4	34634713	34635113	401	208	24	53.46	5.7	70.18
Mcart1-Gm829	4	45485044	45487094	2051	279	114	94.85	4.32	50
Prkaa2-Ppap2b	4	104782759	104783499	741	151	39	50.01	3.83	73.86
BC008163-Fgr	4	132517409	132518270	862	435	56	57.43	3.71	61.11
Tardbp-Gm572	4	148001143	148001993	851	380	60	73.12	4.67	61.9
Slc25a33-Spsb1	4	149250785	149252888	2104	1793	117	69.88	4.16	57.58
BC046331-Lrrc47	4	153384839	153385843	1005	736	55	70.17	5.02	59.38
Tprgl-Arhgef16	4	153535456	153536063	608	318	32	64.44	5.9	62.75
Msx1-Stx18	5	38429731	38430747	1017	283	53	56.86	3.92	61.54
A230054D04Rik-Cpeb2	5	42236005	42236943	939	230	56	84	4.41	41.67

Ppargc1a-Dhx15	5	52260635	52261191	557	275	31	50.45	5.5	73.93
Atp8a1-Grxcr1	5	68274728	68275503	776	361	42	65.44	4.18	66.67
Pdk4-Dync1i1	6	5447040	5447631	592	251	31	51.56	4.18	73.17
Repin1-Zfp775	6	48550595	48551738	1144	255	66	54.46	2.96	66.67
Cpvl-Chn2	6	54214699	54215790	1092	433	56	59.09	3.2	60.22
Gpr175-4933427D06Rik	6	88874915	88876187	1273	772	71	50.55	3.39	74.24
Foxp1-Eif4e3	6	99435648	99436469	822	703	47	55.07	3.13	63.45
Phc1-Rimklb	6	122290264	122291012	749	642	44	51	3.29	74.54
Plcz1-Capza3-Plekha5	6	140286601	140287401	801	250	44	88.67	5.68	22.22
Gltscr1-Zfp541	7	16587003	16587749	747	265	42	57.83	3.92	61.76
Itpkc-Adck4	7	28013642	28014440	799	250	48	64.83	3.8	63.27
Sars2-Nfkbib	7	29541556	29541960	405	283	19	54.2	5.3	64.78
2610034B18Rik-Zfp710	7	87168542	87169804	1263	291	75	53.42	3.71	69.77
Il27-Nupr1	7	133750509	133751217	709	426	40	63.1	4.02	65.52
Brwd2-Fgfr2	7	136921790	136923080	1291	692	81	79.01	4.25	43.75
Ikbkb-Plat	8	23817126	23817980	855	179	60	57.14	3.35	58.77
Ap3m2-Myst3	8	23968772	23969446	675	558	33	62.29	4.7	62.69
Plekha2-Tacc1	8	26226885	26227277	393	256	21	54.62	4.39	66.67
Wdr17-Gpm6a	8	55809909	55810886	978	331	62	55.58	3.17	63.97
Brd7-Nkd1	8	91019371	91020547	1177	860	71	72.16	4.66	53.85
Fa2h-Wdr59	8	113962865	113963436	572	269	27	51.18	4.94	72.77
Gan-Plcg2	8	119784066	119784523	458	333	26	52.21	4.24	72.25
1810063B05Rik-Tomm20	8	129183401	129185775	2375	936	139	51.52	2.9	73.3
1810063B05Rik-Tomm20	8	129216780	129218287	1508	398	93	58.64	2.66	58.76
Pgr-Maml2	9	13217508	13217990	483	203	26	57.16	6.36	59.82
5730601F06Rik-2210010B09Rik	9	20339457	20339918	462	173	21	56.41	6.37	62.9
5730601F06Rik-2210010B09Rik	9	20348373	20349786	1414	296	81	92.56	4.55	25
Pigb-Rab27a	9	72891743	72892479	737	407	41	59.1	4.53	60.87
Dusp7-Rpl29	9	106279681	106281260	1580	347	96	52.01	2.47	73.71
Hhatl-Ccdc13	9	121716186	121718594	2409	130	136	67.44	2.65	63.41
BC023829-OTTMUSG00000017827	X	67730413	67731060	648	319	41	72.21	4.63	56
Nap1l3-3110007F17Rik	X	120118768	120119302	535	260	33	56.31	4.54	62.5
Vmn2r121-4932411N23Rik	X	121306897	121309002	2106	703	232	77.76	3.6	38.89
Mcart6-BC031748	X	133583357	133583785	429	170	19	50.61	6.37	73.89
Zfy2-Sry	Y	1751651	1752336	686	329	42	55.07	4.86	63.89
Rbmy1a1--	Y	2779565	2784511	4947	839	757	55.99	2.92	64.12
Rbmy1a1--	Y	2796136	2797472	1337	173	114	59.44	2.77	63.64
Rbmy1a1--	Y	2850317	2854766	4450	1759	371	102.51	2.78	50
Rbmy1a1--	Y	2856927	2860188	3262	2607	462	190.56	3.86	0
Rbmy1a1--	Y	2860289	2861633	1345	373	156	84.07	3.73	45.45

Supplementary Table 3. MACS analysis of H4K5ac-enriched genomic regions in control over FC

Gene ID	Chromosome	Start	End	Length	Summit	Tags	- 10*log10 (*P*-value)	Fold enrichment	FDR (%)
2410017P07Rik	10	41528630	41529624	995	613	44	60.26	4.46	100
5730410E15Rik	15	44595421	44596381	961	212	47	50.95	3.42	100
Ankrd17	5	90671255	90672548	1294	515	53	50.7	3.02	100
Bst1	5	44233119	44234203	1085	792	51	50.03	3.52	100
Btaf1	19	37031846	37033506	1661	524	71	61.85	3.21	100
Btaf1	19	37045022	37046291	1270	896	58	78.48	3.8	100
Deaf1	7	148506807	148507974	1168	796	49	59.49	3.63	100
Dennd1b	1	140905883	140906797	915	357	38	54.61	4.04	100
Dkk3	7	119275713	119276798	1086	455	50	68.31	3.29	100
Dscr3	16	94741032	94742367	1336	980	66	74.59	3.16	100
Epha6	16	59684689	59686610	1922	453	79	93.68	4.2	100
Ephb2	4	136282610	136283375	766	482	33	51.55	4.52	100
Exoc4	6	33380654	33381980	1327	453	55	53.28	3.55	100
Gnaq	19	16431321	16433991	2671	1712	135	55.04	2.85	100
Gnas	2	174168358	174169458	1101	678	45	50.57	3.56	100
Hspa12a	19	58921296	58923287	1992	1534	84	51.32	3.26	100
Ide	19	37352560	37354564	2005	1399	97	53.77	3.02	100
Ide	19	37385073	37386814	1742	869	84	79.23	3.66	100
ints6	14	63369309	63370031	723	288	31	52.43	4.2	100
Ipo11	13	107668702	107669735	1034	831	45	71.89	4.04	100
Klf11	12	25343343	25344561	1219	780	56	60.78	3.29	100
Klhl7	5	23621852	23622907	1056	647	45	53.74	3.99	100
Loxl2	14	70045877	70048904	3028	1129	151	62.3	2.51	100
Loxl2	14	70084857	70087627	2771	1833	156	58.4	2.99	100
Loxl2	14	70088240	70090180	1941	221	102	78.18	3.49	100
Msi2	11	88408110	88408902	793	434	32	52.64	4.13	100
Mtap2	1	66274228	66275231	1004	397	44	50.71	3.9	100
Mtpn	6	35460812	35462059	1248	372	57	58.86	3.32	100
Nav3	10	109227338	109228558	1221	276	55	83.74	4.1	100
Nckap1	2	80401533	80402777	1245	240	47	51.99	3.29	100
Nfib	4	81976124	81977343	1220	445	55	75.94	4.01	100
Oxr1	15	41627891	41629149	1259	432	46	52.87	3.59	100
Pbrm1	14	31833037	31834187	1151	570	62	58.57	3.49	100
Pdpk1	17	24252797	24253831	1035	720	44	51.25	3.48	100
Pias1	9	62779969	62781116	1148	560	58	58.47	3.63	100
Pigu	2	155145926	155147526	1601	1190	67	53.93	3.7	100
Pip5k1b	19	24525744	24527536	1793	493	90	57.11	3.35	100
Ptk2	15	73065046	73066215	1170	376	51	61.8	3.4	100
Rasal2	1	159221207	159221896	690	481	31	52.12	4.24	100
Rasal2	1	159228370	159229711	1342	848	56	51.93	4.65	100
Rfx3	19	28070549	28071687	1139	212	49	52.39	3.1	100
Slk	19	47661127	47662789	1663	382	71	53.31	3.73	100
Tcf4	18	69814982	69816327	1346	858	74	50.32	3.25	100
Tmtc1	6	148307275	148308871	1597	207	72	65.73	2.67	100
Tspan9	6	128044056	128047098	3043	586	150	51.3	2.85	100

Usp25	16	77033908	77036072	2165	690	113	56.84	3.06	100
Wdr42a	1	174091043	174094034	2992	644	136	80.58	2.92	100
Epb4.1l2-C030003D03Rik	10	25259332	25260314	983	576	42	62.27	4.8	100
Ddo-9030224M15Rik	10	40370772	40373525	2754	2404	126	68.55	2.79	100
Tfam-Ube2d1	10	70701096	70702130	1035	537	45	56.2	4.79	100
Zdhhc17-Osbpl8	10	110533915	110534983	1069	507	52	52.34	3.91	100
Abcb5-Itgb8	12	120223418	120224828	1411	865	58	79.65	4.52	100
plk2-Actbl2	13	111374917	111376424	1508	1026	69	71.72	3.15	100
D930020E02Rik-Entpd4	14	69934402	69935377	976	442	37	52.96	4.68	100
4932438H23Rik-Olig2	16	91081805	91082642	838	319	40	52.29	4.2	100
T-Pde10a	17	8678930	8681107	2178	570	103	53.93	3.08	100
Dtna-Mapre2	18	23960998	23962346	1349	441	68	62.25	3.18	100
Zfp608-Gramd3	18	55155607	55159639	4033	802	187	67.12	2.73	100
Ide-Kif11	19	37448753	37450566	1814	1012	96	56.08	3.1	100
Srp14-Bmf	2	118336620	118337427	808	450	33	55.01	4.13	100
Myef2-Slc12a1	2	124946238	124948271	2034	1494	88	50.16	2.43	100
Gm1527-Evi1	3	29588082	29589155	1074	207	45	65.83	3.72	100
Fbxw7-Pet112l	3	85333843	85335721	1879	514	79	52.52	3.52	100
Slc25a44-Sema4a	3	88229742	88231166	1425	572	69	53.36	2.92	100
1810037I17Rik-Usp53	3	122634200	122635145	946	478	36	52.18	5.5	100
Zfp292-Cga	4	34831735	34833570	1836	453	83	55.87	3.66	100
Rnf38-Melk	4	44248398	44249385	988	561	45	52.3	4.41	100
Oma1-Dab1	4	103341495	103342289	795	208	34	60.67	4.95	100
Oma1-Dab1	4	103454290	103455426	1137	732	53	60.26	3.5	100
Sez6l-Adrbk2	5	113336575	113337752	1178	652	51	77.87	4.79	100
Adrbk2-Crybb2	5	113477929	113479155	1227	358	56	55.75	3.06	100
Luzp2-Siglech	7	63000256	63001196	941	580	40	68.58	5.23	100
EG630138-Clpb	7	108766673	108768895	2223	1091	124	76.12	3.28	100
Zmat4-1810011O10Rik	8	25330818	25332125	1308	496	55	63.6	3.56	100
Apoa5-Cadm1	9	46543451	46545616	2166	562	111	50.7	2.56	100
Senp8-Nr2e3	9	59603009	59604012	1004	559	44	73.82	4.52	100
Rbm3-Tbc1d25	X	7722960	7723790	831	205	37	53.13	3.75	100

Supplementary Table 4. DAVID analysis of genes differentially acetylated for H4K5 in FC over control as identified by MACS

Category	Term	Count	%	*P*-value	List Total	Pop Hits	Pop Total	Fold Enrichment	Bonferroni	Benjamini	FDR
GOTERM_CC_FAT	GO:0045202~synapse	11	10.48	1.52E-05	74	319	12504	5.83	0.00	0.00	0.02
GOTERM_CC_FAT	GO:0000267~cell fraction	13	12.38	1.57E-04	74	596	12504	3.69	0.03	0.01	0.19
SP_PIR_KEYWORDS	antiport	4	3.81	5.13E-04	98	29	17854	25.13	0.08	0.08	0.62
GOTERM_CC_FAT	GO:0005624~membrane fraction	11	10.48	7.19E-04	74	510	12504	3.64	0.11	0.04	0.87
GOTERM_CC_FAT	GO:0005626~insoluble fraction	11	10.48	9.38E-04	74	528	12504	3.52	0.15	0.04	1.14
INTERPRO	IPR011993:Pleckstrin homology-type	8	7.62	0.0012	100	289	17763	4.92	0.30	0.30	1.56
GOTERM_CC_FAT	GO:0044456~synapse part	7	6.67	0.0015	74	212	12504	5.58	0.22	0.05	1.77
SP_PIR_KEYWORDS	Endocytosis	5	4.76	0.0017	98	93	17854	9.79	0.23	0.13	1.98
GOTERM_BP_FAT	GO:0050808~synapse organization	4	3.81	0.0024	75	49	13588	14.79	0.86	0.86	3.61
SP_PIR_KEYWORDS	alternative splicing	38	36.19	0.0030	98	4481	17854	1.54	0.39	0.15	3.58
GOTERM_MF_FAT	GO:0015491~cation:cation antiporter activity	3	2.86	0.0032	77	15	13288	34.51	0.52	0.52	4.06
GOTERM_BP_FAT	GO:0055080~cation homeostasis	6	5.71	0.0033	75	184	13588	5.91	0.93	0.74	4.92
GOTERM_BP_FAT	GO:0007416~synaptogenesis	3	2.86	0.0033	75	16	13588	33.97	0.94	0.60	5.03
GOTERM_CC_FAT	GO:0030424~axon	5	4.76	0.0035	74	107	12504	7.90	0.45	0.09	4.19
GOTERM_BP_FAT	GO:0006897~endocytosis	6	5.71	0.0036	75	188	13588	5.78	0.95	0.52	5.38
GOTERM_BP_FAT	GO:0010324~membrane invagination	6	5.71	0.0036	75	188	13588	5.78	0.95	0.52	5.38
GOTERM_MF_FAT	GO:0015297~antiporter activity	4	3.81	0.0038	77	55	13288	12.55	0.58	0.35	4.77
GOTERM_BP_FAT	GO:0042592~homeostatic process	10	9.52	0.0043	75	584	13588	3.10	0.97	0.51	6.43
GOTERM_BP_FAT	GO:0006811~ion transport	11	10.48	0.0050	75	712	13588	2.80	0.98	0.50	7.50
GOTERM_CC_FAT	GO:0005794~Golgi apparatus	11	10.48	0.0060	74	682	12504	2.73	0.64	0.14	7.10
UP_SEQ_FEATURE	domain:PH	6	5.71	0.0064	94	202	16021	5.06	0.98	0.98	9.05
BIOCARTA	m_appPathway:Generation of amyloid b-peptide by PS1	2	1.90	0.0068	3	4	1171	195.17	0.03	0.03	2.93
GOTERM_BP_FAT	GO:0009100~glycoprotein metabolic process	5	4.76	0.0084	75	147	13588	6.16	1.00	0.63	12.22
GOTERM_BP_FAT	GO:0043062~extracellular structure organization	5	4.76	0.0088	75	149	13588	6.08	1.00	0.60	12.76
GOTERM_MF_FAT	GO:0015298~solute:cation antiporter activity	3	2.86	0.0089	77	25	13288	20.71	0.87	0.49	10.82
SP_PIR_KEYWORDS	phosphoprotein	47	44.76	0.0094	98	6311	17854	1.36	0.78	0.32	10.76
SP_PIR_KEYWORDS	ion transport	9	8.57	0.0094	98	543	17854	3.02	0.78	0.26	10.83
SP_PIR_KEYWORDS	golgi apparatus	9	8.57	0.0105	98	553	17854	2.97	0.82	0.25	11.94
GOTERM_CC_FAT	GO:0042734~presynaptic membrane	3	2.86	0.0115	74	28	12504	18.10	0.86	0.22	13.16
GOTERM_BP_FAT	GO:0055085~transmembrane transport	8	7.62	0.0124	75	460	13588	3.15	1.00	0.68	17.54
GOTERM_BP_FAT	GO:0006874~cellular calcium ion homeostasis	4	3.81	0.0134	75	91	13588	7.96	1.00	0.67	18.73
GOTERM_CC_FAT	GO:0043005~neuron projection	6	5.71	0.0141	74	245	12504	4.14	0.91	0.23	15.92
UP_SEQ_FEATURE	splice variant	37	35.24	0.0142	94	4448	16021	1.42	1.00	0.98	18.94
SP_PIR_KEYWORDS	nucleotide-binding	17	16.19	0.0144	98	1631	17854	1.90	0.90	0.28	16.08
GOTERM_BP_FAT	GO:0055074~calcium ion homeostasis	4	3.81	0.0150	75	95	13588	7.63	1.00	0.68	20.78
INTERPRO	IPR001849:Pleckstrin homology	6	5.71	0.0154	100	261	17763	4.08	0.99	0.91	18.82
GOTERM_BP_FAT	GO:0016044~membrane organization	6	5.71	0.0163	75	272	13588	4.00	1.00	0.67	22.32
GOTERM_BP_FAT	GO:0006875~cellular metal ion homeostasis	4	3.81	0.0172	75	100	13588	7.25	1.00	0.67	23.45
SP_PIR_KEYWORDS	cytoplasm	26	24.76	0.0182	98	3029	17854	1.56	0.95	0.31	19.91
GOTERM_CC_FAT	GO:0042995~cell projection	9	8.57	0.0185	74	575	12504	2.64	0.96	0.27	20.35
INTERPRO	IPR012680:Laminin G, subdomain 2	3	2.86	0.0199	100	39	17763	13.66	1.00	0.88	23.69
GOTERM_BP_FAT	GO:0055065~metal ion homeostasis	4	3.81	0.0201	75	106	13588	6.84	1.00	0.70	26.80
GOTERM_BP_FAT	GO:0048666~neuron development	6	5.71	0.0214	75	292	13588	3.72	1.00	0.69	28.33
GOTERM_BP_FAT	GO:0030182~neuron differentiation	7	6.67	0.0214	75	399	13588	3.18	1.00	0.67	28.34
GOTERM_BP_FAT	GO:0050801~ion homeostasis	6	5.71	0.0217	75	293	13588	3.71	1.00	0.65	28.65

INTERPRO	IPR015447:Neurexin	2	1.90	0.0221	100	4	17763	88.82	1.00	0.82	25.94
GOTERM_CC_FAT	GO:0044459~plasma membrane part	17	16.19	0.0244	74	1633	12504	1.76	0.98	0.31	25.93
GOTERM_MF_FAT	GO:0015300~solute:solute antiporter activity	3	2.86	0.0251	77	43	13288	12.04	1.00	0.77	27.76
SMART	SM00233:PH	6	5.71	0.0262	60	261	9131	3.50	0.91	0.91	25.07
GOTERM_CC_FAT	GO:0005856~cytoskeleton	13	12.38	0.0283	74	1122	12504	1.96	0.99	0.33	29.51
UP_SEQ_FEATURE	domain:Chromo 2	2	1.90	0.0287	94	5	16021	68.17	1.00	1.00	34.81
SP_PIR_KEYWORDS	synapse	5	4.76	0.0289	98	213	17854	4.28	0.99	0.41	29.85
GOTERM_MF_FAT	GO:0031420~alkali metal ion binding	5	4.76	0.0303	77	206	13288	4.19	1.00	0.76	32.57
INTERPRO	IPR006576:BRK domain	2	1.90	0.0330	100	6	17763	59.21	1.00	0.87	36.26
UP_SEQ_FEATURE	propeptide:N-terminal propeptide	2	1.90	0.0343	94	6	16021	56.81	1.00	0.99	40.16
GOTERM_BP_FAT	GO:0055002~striated muscle cell development	3	2.86	0.0349	75	54	13588	10.07	1.00	0.80	42.14
INTERPRO	IPR001331:Guanine-nucleotide dissociation stimulator, CDC24, conserved site	3	2.86	0.0353	100	53	17763	10.05	1.00	0.84	38.24
GOTERM_CC_FAT	GO:0014069~postsynaptic density	3	2.86	0.0357	74	51	12504	9.94	1.00	0.37	35.73
GOTERM_BP_FAT	GO:0030005~cellular di-, tri-valent inorganic cation homeostasis	4	3.81	0.0366	75	134	13588	5.41	1.00	0.80	43.70
GOTERM_BP_FAT	GO:0045931~positive regulation of mitotic cell cycle	2	1.90	0.0375	75	7	13588	51.76	1.00	0.79	44.51
SMART	SM00592:BRK	2	1.90	0.0382	60	6	9131	50.73	0.97	0.82	34.45
SP_PIR_KEYWORDS	cytoskeleton	8	7.62	0.0395	98	583	17854	2.50	1.00	0.48	38.57
GOTERM_CC_FAT	GO:0005583~fibrillar collagen	2	1.90	0.0402	74	7	12504	48.28	1.00	0.39	39.30
UP_SEQ_FEATURE	domain:DH	3	2.86	0.0419	94	56	16021	9.13	1.00	0.99	46.71
GOTERM_BP_FAT	GO:0032989~cellular component morphogenesis	6	5.71	0.0423	75	351	13588	3.10	1.00	0.82	48.63
GOTERM_BP_FAT	GO:0007191~activation of adenylate cyclase activity by dopamine receptor signaling	2	1.90	0.0428	75	8	13588	45.29	1.00	0.80	48.99
GOTERM_BP_FAT	GO:0055001~muscle cell development	3	2.86	0.0436	75	61	13588	8.91	1.00	0.80	49.65
INTERPRO	IPR004709:Na+/H+ exchanger, subfamily	2	1.90	0.0437	100	8	17763	44.41	1.00	0.86	45.15
INTERPRO	IPR018406:Na+/H+ exchanger, conserved region	2	1.90	0.0437	100	8	17763	44.41	1.00	0.86	45.15
GOTERM_MF_FAT	GO:0032553~ribonucleotide binding	17	16.19	0.0452	77	1796	13288	1.63	1.00	0.83	44.74
GOTERM_MF_FAT	GO:0032555~purine ribonucleotide binding	17	16.19	0.0452	77	1796	13288	1.63	1.00	0.83	44.74
GOTERM_BP_FAT	GO:0055066~di-, tri-valent inorganic cation homeostasis	4	3.81	0.0453	75	146	13588	4.96	1.00	0.80	51.06
UP_SEQ_FEATURE	domain:Chromo 1	2	1.90	0.0455	94	8	16021	42.61	1.00	0.99	49.57
SP_PIR_KEYWORDS	atp-binding	13	12.38	0.0459	98	1287	17854	1.84	1.00	0.50	43.37
GOTERM_BP_FAT	GO:0048878~chemical homeostasis	6	5.71	0.0486	75	365	13588	2.98	1.00	0.81	53.61
INTERPRO	IPR018422:Cation/H+ exchanger, conserved region	2	1.90	0.0491	100	9	17763	39.47	1.00	0.86	49.12
INTERPRO	IPR004837:Sodium/calcium exchanger membrane region	2	1.90	0.0491	100	9	17763	39.47	1.00	0.86	49.12
GOTERM_MF_FAT	GO:0005216~ion channel activity	6	5.71	0.0494	77	349	13288	2.97	1.00	0.81	47.74
GOTERM_BP_FAT	GO:0030003~cellular cation homeostasis	4	3.81	0.0500	75	152	13588	4.77	1.00	0.80	54.65
GOTERM_MF_FAT	GO:0015385~sodium:hydrogen antiporter activity	2	1.90	0.0503	77	9	13288	38.35	1.00	0.77	48.40
GOTERM_MF_FAT	GO:0005451~monovalent cation:hydrogen antiporter activity	2	1.90	0.0503	77	9	13288	38.35	1.00	0.77	48.40
INTERPRO	IPR000219:Dbl homology (DH) domain	3	2.86	0.0510	100	65	17763	8.20	1.00	0.84	50.48
GOTERM_BP_FAT	GO:0007423~sensory organ development	5	4.76	0.0515	75	257	13588	3.52	1.00	0.80	55.69
GOTERM_BP_FAT	GO:0042472~inner ear morphogenesis	3	2.86	0.0515	75	67	13588	8.11	1.00	0.79	55.75
GOTERM_MF_FAT	GO:0005085~guanyl-nucleotide exchange factor activity	4	3.81	0.0520	77	147	13288	4.70	1.00	0.74	49.53
GOTERM_MF_FAT	GO:0022838~substrate specific channel activity	6	5.71	0.0551	77	360	13288	2.88	1.00	0.73	51.59
GOTERM_MF_FAT	GO:0030695~GTPase regulator activity	6	5.71	0.0556	77	361	13288	2.87	1.00	0.70	51.94
UP_SEQ_FEATURE	domain:EGF-like 3	3	2.86	0.0563	94	66	16021	7.75	1.00	0.99	57.31
UP_SEQ_FEATURE	domain:EGF-like 12	2	1.90	0.0566	94	10	16021	34.09	1.00	0.99	57.51
UP_SEQ_FEATURE	domain:Fibrillar collagen NC1	2	1.90	0.0566	94	10	16021	34.09	1.00	0.99	57.51
GOTERM_MF_FAT	GO:0022803~passive transmembrane transporter activity	6	5.71	0.0578	77	365	13288	2.84	1.00	0.68	53.33
GOTERM_MF_FAT	GO:0015267~channel activity	6	5.71	0.0578	77	365	13288	2.84	1.00	0.68	53.33
GOTERM_MF_FAT	GO:0060589~nucleoside-triphosphatase regulator activity	6	5.71	0.0589	77	367	13288	2.82	1.00	0.66	54.03
GOTERM_BP_FAT	GO:0051240~positive regulation of multicellular organismal process	4	3.81	0.0593	75	163	13588	4.45	1.00	0.82	60.98
INTERPRO	IPR000885:Fibrillar collagen, C-terminal	2	1.90	0.0596	100	11	17763	32.30	1.00	0.85	56.22
INTERPRO	IPR015721:Rho GTP exchange factor	2	1.90	0.0596	100	11	17763	32.30	1.00	0.85	56.22
GOTERM_MF_FAT	GO:0017076~purine nucleotide binding	17	16.19	0.0617	77	1871	13288	1.57	1.00	0.65	55.80

UP_SEQ_FEATURE	domain:EGF-like 11	2	1.90	0.0621	94	11	16021	30.99	1.00	0.98	61.00
GOTERM_MF_FAT	GO:0005089~Rho guanyl-nucleotide exchange factor activity	3	2.86	0.0623	77	71	13288	7.29	1.00	0.63	56.13
SP_PIR_KEYWORDS	cell junction	6	5.71	0.0625	98	392	17854	2.79	1.00	0.58	54.19
GOTERM_BP_FAT	GO:0040014~regulation of multicellular organism growth	3	2.86	0.0629	75	75	13588	7.25	1.00	0.83	63.27
GOTERM_BP_FAT	GO:0042471~ear morphogenesis	3	2.86	0.0644	75	76	13588	7.15	1.00	0.83	64.15
INTERPRO	IPR006153:Cation/H+ exchanger	2	1.90	0.0649	100	12	17763	29.61	1.00	0.85	59.38
GOTERM_CC_FAT	GO:0005886~plasma membrane	24	22.86	0.0654	74	2906	12504	1.40	1.00	0.53	56.14
SMART	SM00325:RhoGEF	3	2.86	0.0659	60	65	9131	7.02	1.00	0.87	52.29
GOTERM_MF_FAT	GO:0005246~calcium channel regulator activity	2	1.90	0.0665	77	12	13288	28.76	1.00	0.63	58.61
UP_SEQ_FEATURE	domain:EGF-like 15	2	1.90	0.0675	94	12	16021	28.41	1.00	0.98	64.20
GOTERM_BP_FAT	GO:0007215~glutamate signaling pathway	2	1.90	0.0686	75	13	13588	27.87	1.00	0.84	66.52
SMART	SM00038:COLFI	2	1.90	0.0689	60	11	9131	27.67	1.00	0.80	53.91
GOTERM_BP_FAT	GO:0007610~behavior	6	5.71	0.0696	75	405	13588	2.68	1.00	0.83	67.10
GOTERM_BP_FAT	GO:0019637~organophosphate metabolic process	4	3.81	0.0711	75	176	13588	4.12	1.00	0.83	67.91
UP_SEQ_FEATURE	domain:EGF-like 14	2	1.90	0.0729	94	13	16021	26.22	1.00	0.98	67.13
UP_SEQ_FEATURE	domain:MAM	2	1.90	0.0729	94	13	16021	26.22	1.00	0.98	67.13
INTERPRO	IPR003585:Neurexin/syndecan/glycophorin C	2	1.90	0.0753	100	14	17763	25.38	1.00	0.87	65.05
PIR_SUPERFAMILY	PIRSF005557:sialyltransferase	2	1.90	0.0759	41	16	8136	24.80	0.98	0.98	52.92
GOTERM_BP_FAT	GO:0046578~regulation of Ras protein signal transduction	4	3.81	0.0759	75	181	13588	4.00	1.00	0.84	70.38
GOTERM_BP_FAT	GO:0003001~generation of a signal involved in cell-cell signaling	3	2.86	0.0767	75	84	13588	6.47	1.00	0.84	70.73
GOTERM_BP_FAT	GO:0048667~cell morphogenesis involved in neuron differentiation	4	3.81	0.0769	75	182	13588	3.98	1.00	0.83	70.86
GOTERM_MF_FAT	GO:0022836~gated channel activity	5	4.76	0.0772	77	281	13288	3.07	1.00	0.66	64.26
UP_SEQ_FEATURE	domain:EGF-like 13	2	1.90	0.0783	94	14	16021	24.35	1.00	0.98	69.83
GOTERM_BP_FAT	GO:0035023~regulation of Rho protein signal transduction	3	2.86	0.0798	75	86	13588	6.32	1.00	0.83	72.24
PIR_SUPERFAMILY	PIRSF005557:Sialyl_trans	2	1.90	0.0804	41	17	8136	23.35	0.98	0.87	55.09
GOTERM_MF_FAT	GO:0005088~Ras guanyl-nucleotide exchange factor activity	3	2.86	0.0817	77	83	13288	6.24	1.00	0.66	66.44
GOTERM_BP_FAT	GO:0048839~inner ear development	3	2.86	0.0830	75	88	13588	6.18	1.00	0.84	73.70
GOTERM_BP_FAT	GO:0015672~monovalent inorganic cation transport	5	4.76	0.0831	75	303	13588	2.99	1.00	0.83	73.74
UP_SEQ_FEATURE	domain:EGF-like 10	2	1.90	0.0837	94	15	16021	22.72	1.00	0.98	72.31
GOTERM_BP_FAT	GO:0051146~striated muscle cell differentiation	3	2.86	0.0847	75	89	13588	6.11	1.00	0.83	74.40
SP_PIR_KEYWORDS	coiled coil	15	14.29	0.0849	98	1732	17854	1.58	1.00	0.67	65.83
SMART	SM00294:4.1m	2	1.90	0.0868	60	14	9131	21.74	1.00	0.80	62.69
GOTERM_BP_FAT	GO:0000902~cell morphogenesis	5	4.76	0.0878	75	309	13588	2.93	1.00	0.83	75.74
GOTERM_MF_FAT	GO:0008092~cytoskeletal protein binding	6	5.71	0.0881	77	414	13288	2.50	1.00	0.67	69.33
INTERPRO	IPR012163:Sialyltransferase	2	1.90	0.0907	100	17	17763	20.90	1.00	0.90	72.10
GOTERM_CC_FAT	GO:0012505~endomembrane system	7	6.67	0.0918	74	535	12504	2.21	1.00	0.64	69.02
GOTERM_BP_FAT	GO:0030001~metal ion transport	6	5.71	0.0929	75	442	13588	2.46	1.00	0.84	77.72
SP_PIR_KEYWORDS	glycosyltransferase	4	3.81	0.0943	98	199	17854	3.66	1.00	0.68	69.83
INTERPRO	IPR000998:MAM	2	1.90	0.0958	100	18	17763	19.74	1.00	0.89	74.12
SP_PIR_KEYWORDS	sh3 domain	4	3.81	0.0997	98	204	17854	3.57	1.00	0.68	71.95
INTERPRO	IPR000742:EGF-like, type 3	4	3.81	0.0999	100	199	17763	3.57	1.00	0.89	75.67

Summary of functional annotation data for genes differentially acetylated for H4K5 in FC over control (FC) using DAVID.

Supplementary Table 5. DAVID analysis of genes differentially acetylated for H4K5 in control over FC as identified by MACS

Category	Term	Count	%	*P*-value	List Total	Pop Hits	Pop Total	Fold Enrichment	Bonferroni	Benjamini	FDR
GOTERM_MF_FAT	GO:0000166~nucleotide binding	14	34.15	1.25E-04	28	2183	13288	3.04	0.01	0.01	0.13
SP_PIR_KEYWORDS	alternative splicing	21	51.22	3.89E-04	40	4481	17854	2.09	0.03	0.03	0.42
GOTERM_MF_FAT	GO:0032553~ribonucleotide binding	12	29.27	4.33E-04	28	1796	13288	3.17	0.03	0.02	0.45
GOTERM_MF_FAT	GO:0032555~purine ribonucleotide binding	12	29.27	4.33E-04	28	1796	13288	3.17	0.03	0.02	0.45
GOTERM_MF_FAT	GO:0017076~purine nucleotide binding	12	29.27	6.20E-04	28	1871	13288	3.04	0.04	0.02	0.65
UP_SEQ_FEATURE	splice variant	21	51.22	0.0010	39	4448	16021	1.94	0.19	0.19	1.29
GOTERM_MF_FAT	GO:0005524~ATP binding	10	24.39	0.0016	28	1443	13288	3.29	0.11	0.03	1.62
GOTERM_MF_FAT	GO:0032559~adenyl ribonucleotide binding	10	24.39	0.0017	28	1460	13288	3.25	0.12	0.02	1.76
GOTERM_BP_FAT	GO:0021954~central nervous system neuron development	3	7.32	0.0024	31	33	13588	39.85	0.67	0.67	3.35
GOTERM_MF_FAT	GO:0030554~adenyl nucleotide binding	10	24.39	0.0024	28	1535	13288	3.09	0.16	0.03	2.49
GOTERM_MF_FAT	GO:0001883~purine nucleoside binding	10	24.39	0.0026	28	1548	13288	3.07	0.17	0.03	2.64
GOTERM_MF_FAT	GO:0001882~nucleoside binding	10	24.39	0.0027	28	1558	13288	3.05	0.18	0.02	2.76
GOTERM_BP_FAT	GO:0021953~central nervous system neuron differentiation	3	7.32	0.0038	31	42	13588	31.31	0.83	0.59	5.34
SP_PIR_KEYWORDS	phosphoprotein	23	56.10	0.0057	40	6311	17854	1.63	0.39	0.22	5.98
GOTERM_MF_FAT	GO:0003677~DNA binding	10	24.39	0.0066	28	1781	13288	2.66	0.39	0.05	6.72
BIOCARTA	m_CCR3Pathway:CCR3 signaling in Eosinophils	3	7.32	0.0067	8	22	1171	19.96	0.28	0.28	6.23
SP_PIR_KEYWORDS	nucleotide-binding	10	24.39	0.0074	40	1631	17854	2.74	0.47	0.19	7.66
GOTERM_BP_FAT	GO:0030182~neuron differentiation	5	12.20	0.0110	31	399	13588	5.49	0.99	0.82	14.55
GOTERM_BP_FAT	GO:0006468~protein amino acid phosphorylation	6	14.63	0.0122	31	640	13588	4.11	1.00	0.76	16.08
GOTERM_BP_FAT	GO:0051129~negative regulation of cellular component organization	3	7.32	0.0178	31	93	13588	14.14	1.00	0.81	22.59
SP_PIR_KEYWORDS	kinase	6	14.63	0.0182	40	707	17854	3.79	0.79	0.32	17.89
INTERPRO	IPR017441:Protein kinase, ATP binding site	5	12.20	0.0188	40	467	17763	4.75	0.90	0.90	19.52
UP_SEQ_FEATURE	active site:Proton acceptor	6	14.63	0.0193	39	662	16021	3.72	0.98	0.86	21.73
GOTERM_BP_FAT	GO:0016310~phosphorylation	6	14.63	0.0193	31	718	13588	3.66	1.00	0.78	24.24
SP_PIR_KEYWORDS	atp-binding	8	19.51	0.0200	40	1287	17854	2.77	0.82	0.29	19.54
INTERPRO	IPR008266:Tyrosine protein kinase, active site	3	7.32	0.0207	40	101	17763	13.19	0.92	0.71	21.31
INTERPRO	IPR000719:Protein kinase, core	5	12.20	0.0221	40	491	17763	4.52	0.93	0.59	22.60
PIR_SUPERFAMILY	PIRSF000666:TyrPK_ephrin_receptor	2	4.88	0.0222	15	13	8136	83.45	0.30	0.30	14.71
PIR_SUPERFAMILY	PIRSF002400:GTP-binding regulatory protein Gs alpha chain	2	4.88	0.0238	15	14	8136	77.49	0.32	0.18	15.75
UP_SEQ_FEATURE	domain:Protein kinase	5	12.20	0.0256	39	476	16021	4.32	1.00	0.83	27.84
GOTERM_BP_FAT	GO:0048666~neuron development	4	9.76	0.0260	31	292	13588	6.00	1.00	0.83	31.30
SP_PIR_KEYWORDS	tyrosine-protein kinase	3	7.32	0.0265	40	116	17854	11.54	0.90	0.32	25.11
UP_SEQ_FEATURE	mutagenesis site	6	14.63	0.0270	39	723	16021	3.41	1.00	0.75	29.16
GOTERM_MF_FAT	GO:0005003~ephrin receptor activity	2	4.88	0.0281	28	14	13288	67.80	0.88	0.19	25.78
INTERPRO	IPR016257:Tyrosine-protein kinase, ephrin receptor	2	4.88	0.0282	40	13	17763	68.32	0.97	0.57	27.94
GOTERM_MF_FAT	GO:0004672~protein kinase activity	5	12.20	0.0289	28	583	13288	4.07	0.89	0.18	26.40
GOTERM_CC_FAT	GO:0031252~cell leading edge	3	7.32	0.0294	31	112	12504	10.80	0.94	0.94	28.00
UP_SEQ_FEATURE	compositionally biased region:Ala-rich	3	7.32	0.0294	39	113	16021	10.91	1.00	0.71	31.30
INTERPRO	IPR001245:Tyrosine protein kinase	3	7.32	0.0303	40	124	17763	10.74	0.97	0.52	29.70
INTERPRO	IPR001426:Receptor tyrosine kinase, class V, conserved site	2	4.88	0.0303	40	14	17763	63.44	0.97	0.46	29.73
INTERPRO	IPR001090:Ephrin receptor, ligand binding	2	4.88	0.0303	40	14	17763	63.44	0.97	0.46	29.73
SP_PIR_KEYWORDS	signal transduction	2	4.88	0.0323	40	15	17854	59.51	0.94	0.33	29.75
GOTERM_BP_FAT	GO:0030030~cell projection organization	4	9.76	0.0326	31	319	13588	5.50	1.00	0.85	37.65
GOTERM_BP_FAT	GO:0021955~central nervous system neuron axonogenesis	2	4.88	0.0326	31	15	13588	58.44	1.00	0.82	37.67
KEGG_PATHWAY	mmu04360:Axon guidance	3	7.32	0.0342	14	131	5738	9.39	0.72	0.72	26.85

INTERPRO	IPR001019:Guanine nucleotide binding protein (G-protein), alpha subunit	2	4.88	0.0346	40	16	17763	55.51	0.98	0.45	33.19
GOTERM_BP_FAT	GO:0050771~negative regulation of axonogenesis	2	4.88	0.0348	31	16	13588	54.79	1.00	0.81	39.61
SMART	SM00615:EPH_lbd	2	4.88	0.0362	25	14	9131	52.18	0.75	0.75	28.34
GOTERM_CC_FAT	GO:0012505~endomembrane system	5	12.20	0.0376	31	535	12504	3.77	0.97	0.84	34.45
UP_SEQ_FEATURE	binding site:ATP	5	12.20	0.0393	39	545	16021	3.77	1.00	0.75	39.58
GOTERM_BP_FAT	GO:0006793~phosphorus metabolic process	6	14.63	0.0393	31	866	13588	3.04	1.00	0.82	43.49
GOTERM_BP_FAT	GO:0006796~phosphate metabolic process	6	14.63	0.0393	31	866	13588	3.04	1.00	0.82	43.49
SMART	SM00219:TyrKc	3	7.32	0.0416	25	124	9131	8.84	0.80	0.55	31.86
GOTERM_BP_FAT	GO:0010171~body morphogenesis	2	4.88	0.0433	31	20	13588	43.83	1.00	0.82	46.77
GOTERM_BP_FAT	GO:0031345~negative regulation of cell projection organization	2	4.88	0.0433	31	20	13588	43.83	1.00	0.82	46.77
GOTERM_BP_FAT	GO:0007212~dopamine receptor signaling pathway	2	4.88	0.0433	31	20	13588	43.83	1.00	0.82	46.77
GOTERM_MF_FAT	GO:0004713~protein tyrosine kinase activity	3	7.32	0.0434	28	164	13288	8.68	0.96	0.24	37.18
GOTERM_CC_FAT	GO:0042995~cell projection	5	12.20	0.0470	31	575	12504	3.51	0.99	0.79	41.17
SP_PIR_KEYWORDS	heterotrimer	2	4.88	0.0512	40	24	17854	37.20	0.99	0.43	43.16
UP_SEQ_FEATURE	compositionally biased region:Ser-rich	4	9.76	0.0607	39	380	16021	4.32	1.00	0.84	54.51
GOTERM_BP_FAT	GO:0007492~endoderm development	2	4.88	0.0621	31	29	13588	30.23	1.00	0.90	59.93
GOTERM_BP_FAT	GO:0007169~transmembrane receptor protein tyrosine kinase signaling pathway	3	7.32	0.0667	31	192	13588	6.85	1.00	0.90	62.64
KEGG_PATHWAY	mmu04020:Calcium signaling pathway	3	7.32	0.0676	14	191	5738	6.44	0.92	0.73	46.65
GOTERM_MF_FAT	GO:0016564~transcription repressor activity	3	7.32	0.0680	28	211	13288	6.75	0.99	0.33	52.13
GOTERM_BP_FAT	GO:0010579~positive regulation of adenylate cyclase activity by G-protein signaling	2	4.88	0.0684	31	32	13588	27.40	1.00	0.89	63.55
GOTERM_BP_FAT	GO:0010578~regulation of adenylate cyclase activity involved in G-protein signaling	2	4.88	0.0684	31	32	13588	27.40	1.00	0.89	63.55
GOTERM_BP_FAT	GO:0007189~activation of adenylate cyclase activity by G-protein signaling pathway	2	4.88	0.0684	31	32	13588	27.40	1.00	0.89	63.55
GOTERM_MF_FAT	GO:0003690~double-stranded DNA binding	2	4.88	0.0688	28	35	13288	27.12	0.99	0.31	52.58
SP_PIR_KEYWORDS	GTP binding	2	4.88	0.0737	40	35	17854	25.51	1.00	0.51	56.14
GOTERM_BP_FAT	GO:0050768~negative regulation of neurogenesis	2	4.88	0.0807	31	38	13588	23.07	1.00	0.91	69.84
GOTERM_CC_FAT	GO:0005834~heterotrimeric G-protein complex	2	4.88	0.0807	31	35	12504	23.05	1.00	0.87	60.44
SP_PIR_KEYWORDS	dna-binding	7	17.07	0.0822	40	1404	17854	2.23	1.00	0.52	60.25
GOTERM_BP_FAT	GO:0050770~regulation of axonogenesis	2	4.88	0.0827	31	39	13588	22.48	1.00	0.91	70.78
GOTERM_BP_FAT	GO:0031175~neuron projection development	3	7.32	0.0831	31	218	13588	6.03	1.00	0.89	70.98
KEGG_PATHWAY	mmu04810:Regulation of actin cytoskeleton	3	7.32	0.0844	14	217	5738	5.67	0.96	0.66	54.72
GOTERM_BP_FAT	GO:0010721~negative regulation of cell development	2	4.88	0.0847	31	40	13588	21.92	1.00	0.89	71.69
GOTERM_BP_FAT	GO:0007507~heart development	3	7.32	0.0864	31	223	13588	5.90	1.00	0.88	72.43
GOTERM_BP_FAT	GO:0007190~activation of adenylate cyclase activity	2	4.88	0.0868	31	41	13588	21.38	1.00	0.87	72.57
GOTERM_BP_FAT	GO:0031281~positive regulation of cyclase activity	2	4.88	0.0888	31	42	13588	20.87	1.00	0.86	73.42
GOTERM_BP_FAT	GO:0045762~positive regulation of adenylate cyclase activity	2	4.88	0.0888	31	42	13588	20.87	1.00	0.86	73.42
GOTERM_BP_FAT	GO:0051349~positive regulation of lyase activity	2	4.88	0.0888	31	42	13588	20.87	1.00	0.86	73.42
GOTERM_MF_FAT	GO:0030528~transcription regulator activity	6	14.63	0.0923	28	1206	13288	2.36	1.00	0.38	63.73
GOTERM_BP_FAT	GO:0010975~regulation of neuron projection development	2	4.88	0.0988	31	47	13588	18.65	1.00	0.88	77.31

Summary of functional annotation data for genes differentially acetylated for H4K5 in control over FC (Control) using DAVID.

Supplementary Table 6. Oligonucleotides used for quantitative RT-PCR

Gene ID	Primer	Sequence	Caller	Condition
Akap6	F_akap6	GCTCATTGCGTCTCACAAAG	MACS	FC
Akap6	R_akap6	ATCTCGGCTTTGATGGTGTC	MACS	FC
Atp8a1	F_atp8a1	ATCCACGTCGGTACAGAACAG	MACS	FC
Atp8a1	R_atp8a1	ATGGTTTCAGGCACTTGGTC	MACS	FC
Col2a1	F_col2a1	GCAGCAAGAGCAAGGAAAAG	MACS	FC
Col2a1	R_col2a1	GGAAAGTCATCTGGACGTTAGC	MACS	FC
Grid1	F_grid1	CCGTCTCAATGATGTCATGC	MACS	FC
Grid1	R_grid1	TGATCCAGGAAGCTTTGGAG	MACS	FC
Kcnd2	F_kcnd2	AATGTGTCGGGAAGCCATAG	MACS	FC
Kcnd2	R_kcnd2	TGGCATTTAAGCTGGATCG	MACS	FC
Kdm2a	F_fbxl11	CGAAGATGCTCTGATTGCTG	MACS	FC
Kdm2a	R_fbxl11	TTATCCCTTTTTGGCCACTG	MACS	FC
Megf11	F_megf11	CTTTCGAGGACCCTTATGTCAG	MACS	FC
Megf11	R_megf11	TGGCAGGCACTCACAGATAC	MACS	FC
Nrxn1	F_nrxn1	ACAGTGGGGATGGAAATGAC	MACS	FC
Nrxn1	R_nrxn1	TTAGCACCGTTTCCCAAGTC	MACS	FC
Ntrk2	F_ntrk2	GTGGCAGAAAACCTTGTAGGAG	MACS	FC
Ntrk2	R_ntrk2	CAGTGAATGGAATGCACCAG	MACS	FC
Rnf220	F_rnf220	TGTGTCTGGCCTCATCTCTG	MACS	FC
Rnf220	R_rnf220	TGGCTGTCAAACAACGCTAC	MACS	FC
Ryr3	F_ryr3	ACACCAAATCCACAGCCTTC	MACS	FC
Ryr3	R_ryr3	TCCCCGTACTTGATTTCTGG	MACS	FC
Serpina9	F_serpina9	GCCTTTAACTCAAACGCTGAC	MACS	FC
Serpina9	R_serpina9	GCGGACTATAAGTTTGGTGGTG	MACS	FC

Oligonucleotide primer sequences used for quantitative RT-PCR validation of cDNA-converted genes.
